# Dissection of the spatial dynamics of biosynthesis, transport, and turnover of major amino acids in tea plants (*Camellia sinensis*)

**DOI:** 10.1093/hr/uhae060

**Published:** 2024-02-19

**Authors:** Shuwei Yu, Mingzhi Zhu, Ping Li, Hao Zuo, Juan Li, Yingying Li, Anqi Peng, Jianan Huang, Alisdair R Fernie, Zhonghua Liu, Jian Zhao

**Affiliations:** Key Laboratory of Tea Science of Ministry of Education, College of Horticulture, Hunan Agricultural University, Changsha 410128, China; Tea Research Institute, Shandong Academy of Agricultural Sciences, Jinan 250000, China; Key Laboratory of Tea Science of Ministry of Education, College of Horticulture, Hunan Agricultural University, Changsha 410128, China; National Research Center of Engineering and Technology for Utilization of Botanical Functional Ingredients & Co-Innovation Center of Education Ministry for Utilization of Botanical Functional Ingredients, Hunan Agricultural University, Changsha 410128, China; National Research Center of Engineering and Technology for Utilization of Botanical Functional Ingredients & Co-Innovation Center of Education Ministry for Utilization of Botanical Functional Ingredients, Hunan Agricultural University, Changsha 410128, China; Key Laboratory of Tea Science of Ministry of Education, College of Horticulture, Hunan Agricultural University, Changsha 410128, China; Biotechnology Center, Anhui Agricultural University, Hefei 230036, China; State Key Laboratory of Tea Plant Biology and Utilization, Anhui Agricultural University, Hefei 230036, China; Key Laboratory of Tea Science of Ministry of Education, College of Horticulture, Hunan Agricultural University, Changsha 410128, China; National Research Center of Engineering and Technology for Utilization of Botanical Functional Ingredients & Co-Innovation Center of Education Ministry for Utilization of Botanical Functional Ingredients, Hunan Agricultural University, Changsha 410128, China; Key Laboratory of Tea Science of Ministry of Education, College of Horticulture, Hunan Agricultural University, Changsha 410128, China; National Research Center of Engineering and Technology for Utilization of Botanical Functional Ingredients & Co-Innovation Center of Education Ministry for Utilization of Botanical Functional Ingredients, Hunan Agricultural University, Changsha 410128, China; Max-Planck-Institute of Molecular Plant Physiology , Am Mühlenberg 1, 14476 Potsdam-Golm, Germany; Key Laboratory of Tea Science of Ministry of Education, College of Horticulture, Hunan Agricultural University, Changsha 410128, China; National Research Center of Engineering and Technology for Utilization of Botanical Functional Ingredients & Co-Innovation Center of Education Ministry for Utilization of Botanical Functional Ingredients, Hunan Agricultural University, Changsha 410128, China; Key Laboratory of Tea Science of Ministry of Education, College of Horticulture, Hunan Agricultural University, Changsha 410128, China; National Research Center of Engineering and Technology for Utilization of Botanical Functional Ingredients & Co-Innovation Center of Education Ministry for Utilization of Botanical Functional Ingredients, Hunan Agricultural University, Changsha 410128, China

## Abstract

High levels of free amino acids (AAs) in tea leaves are crucial for tea flavor and health function; however, the dynamic AA biosynthesis, transport, and turnover in tea plants remain elusive. Here we dissected whole tea plants for these dynamics by assessing AA profiles and transcriptomes of metabolic pathway genes in tea roots, stems, and leaves and revealing their distinctive features with regard to AA synthesis, transport, and degradation/recycling. Nitrogen assimilation dominated in the roots wherein glutamine (Gln), theanine, and arginine (Arg) were actively synthesized. Arg was transported into trunk roots and stems, together with Glu, Gln, and theanine as the major AAs in the xylem sap for long-distance root-to-leaf transport. Transcriptome analysis revealed that genes involved in Arg synthesis were highly expressed in roots, but those for Arg transport and degradation were highly expressed in stems and young leaves, respectively. *CsGSIa* transcripts were found in root meristem cells, root, stem and leaf vascular tissues, and leaf mesophyll where it appeared to participate in AA synthesis, transport, and recycling. Overexpression of *CsGSIa* in tea transgenic hairy roots and knockdown of *CsGSIa* in transgenic hairy roots and tea leaves produced higher and lower Gln and theanine than wild-type roots and leaves, respectively. This study provides comprehensive and new insights into AA metabolism and transport in the whole tea plant.

## Introduction

Nitrogen (N) is a limiting macronutrient for plant growth and other life activities. Plants assimilate N primarily in the form of ammonium (NH_4_^**+**^) that is directly taken up from soils or generated from reduction of nitrate (NO_3_^−^) from soils in their roots. The ammonia is incorporated into glutamate (Glu) to form glutamine (Gln) by glutamine synthetase (GS) and thereafter other amino acids (AAs) [1, 2] (Ruan et al., 2010). Gln synthase (GOGAT) transfers NH_3_ from Gln to 2-oxoglutarate (2OG) thereby generating Glu, whereas glutamate dehydrogenase (GDH) catalyzes the deamination of glutamate and reductive amination of 2OG to form glutamate in nitrogen metabolism in the mitochondria and cytosol [3]. The nitrate assimilation pathway starts with nitrate uptake by root nitrate transporters (NRTs), followed by successive nitrate and nitrite reduction resulting in the generation of NH_4_^**+**^, which is subsequently fixed into AAs [4, 5]. In plant root cells, nitrate reduction into nitrite by nitrate reductase (NR) occurs in the cytosol, and nitrite then enters the plastid for reduction to ammonia by nitrite reductase (NIR).

Among the 21 proteinogenic AAs, arginine (Arg) is a major storage and transport form of organic N in many plants, contributing for example 40–50% of total N or AA pools in developing legume seeds [6, 7]. Arg is also a precursor for polyamines and nitric oxide, which play important roles in signal transduction [8]. Arg is primarily synthesized in the chloroplast via the ornithine (Orn) and citrulline (Cit) pathways, which are tightly regulated by various feedback mechanisms [7, 9]. Orn is synthesized from Glu via a range of acetylated intermediates [7, 10]. Orn depletion is associated with enhanced polyamine metabolism and alterations in plant development [3, 10]. Orn transcarbamoylase (OTC) catalyzes the carbamoylation of Orn to generate Cit, thereafter the N from aspartate (Asp) can be transferred to Cit by argininosuccinate synthase (ASSY) to produce fumarate, which can be cleaved by argininosuccinate lyase (ASL) to generate Arg [3]. Arg can be hydrolyzed by L-arginine ureahydrolase (ARGH) to yield Orn and Urea, which can be further hydrolyzed by Urease into two ammonia (NH_3_), liberating carbon dioxide. NH_3_ then enters into the GS/GOGAT cycle for N re-assimilation [11]. The increased Urease and ARGH activities are prerequisites for N re-assimilation. Orn is also a precursor for proline and Glu synthesis via AA recycling pathways [7, 12]. Arg displays the highest N:C (4:6) ratio among AA molecules, and as such is often considered a major N storage and transport molecule in plants [7, 12]. Various AA transporters, such as the LYSINE HISTIDINE TRANSPORTER1 (LHT1), basic amino acid carriers (BACs), bidirectional AA transporter 1 (AtBAT1), Usually Multiple Acids Move In and out Transporters (UMAMITs) and cationic AA transporters (CATs) have been reported to facilitate high- or low-affinity AA transport on various membrane systems for subcellular or intercellular transport, loading or unloading of AAs from xylem or phloem in long distance transport [13–16] (Dinkeloo *et al.*, 2018).

Tea plant (*Camellia sinensis* (L.) O. Kuntze) prefers to use ammonia as N fertilizer (Ruan *et al.*,2010) [1, 17, 18]. Theanine (γ-glutamylethylamide, Thea) is the most abundant free non-proteinogenic AA, accounting for more than 50–70% of total free AAs and 1–4% of the dry tea leaves [19, 20]. Thea and other AAs contribute both to the umami and sweet tastes of tea infusions and provide many health benefits including mood relaxation, cognitive improvement, and neuronal protection [21–23]. Moreover, total AA content in tea represents one of the most important criteria for tea flavor quality and health functions. The higher the total AA contents in tea leaves, the better tea quality and health functions [20, 24, 25]. However, the dynamic AA biosynthesis, transport, and turnover in tea plants remain elusive partially due to the fact that Thea accounts for most of total free AAs and its biosynthesis and regulations are still not fully understood. Therefore, root N assimilation, transport of AAs from roots to young leaves for metabolism and accumulation in young tea leaves are of great interest for tea scientists to understand. Although N assimilation and AA metabolism are well known in many plants, the presence of Thea at much higher levels in tea roots and leaves than other major AAs complicates the situation in tea plants [20]. While theanine synthetase I (CsTSI) is capable of synthesizing Thea from Glu and ethylamine (EA) [26, 27], cytosolic CsGSIs and a plastidic CsGSIIa were also implied in Thea biosynthesis when heterologously expressed in other plants [28–30]. EA may be derived from alanine (Ala) by the action of alanine decarboxylase (AlaDC) [31]. Most Thea is synthesized in tea roots and subsequently transported to shoot tips [21, 32], with AA transporters being characterized that transport Thea and others AAs [33]. However, fine details regarding the functions of the CsGS/CsTSI enzymes remain elusive due to discrepancies in their differential gene expression and Gln and Thea accumulation patterns [29]. Here, we conducted AA metabolite profiling and transcriptome analyses in various tissues of tea plants to dissect the dynamics of tea N assimilation, transport and metabolism. Our study demonstrates distinctive features replace of AA synthesis, transport and metabolism in tea roots, stems, and leaves, and that Arg, Gln, Glu, and Thea are the major AA forms in tea xylem sap for root–leaf transport, while CsGSIa plays critical roles in the processes operating within these tissues.

## Results

### Distribution of free AAs in tissues of tea plants with or without NH_4_^+^ fed

**Figure 1:**
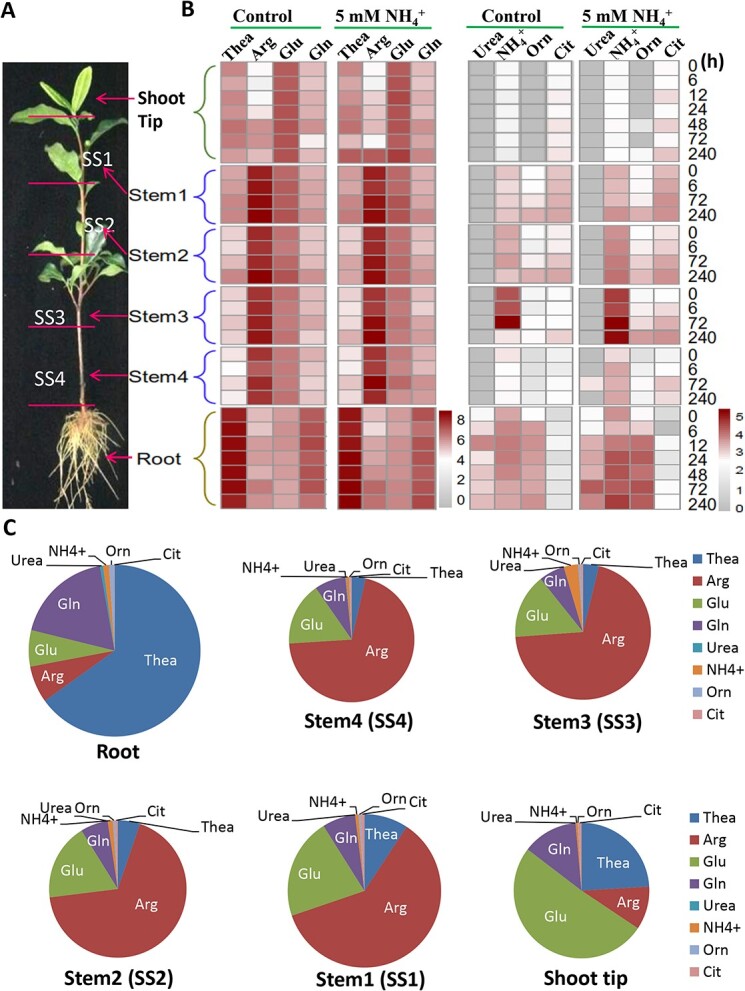
Profiles of the major free AAs and NH_2_-containing metabolites in various tissues of tea plants. (**A**) The representative image of a two-year-old tea seedling grown hydroponically under normal N or 5 mM NH_4_^+^-supplied SK solutions, with dissection of six tissues, SS, stem segments; shoot tip, including the apical bud and the first leaf. (**B**) Profiles of AAs and NH_2_-containing metabolites in the root, stem segments, shoot tip of the tea plant seedling. (**C**) The pie chart analyses of proportions of six major free AAs, urea and ammonium in total NH_2_-containing metabolites in six tea tissues under 5 mM NH_4_^+^-supply condition. Data are from at least three independent experiments with biological replicates. Arg, arginine; Cit, citrulline; Gln, glutamine; Glu, glutamate; NH_4_^+^, ammonium; Orn, ornithine; Thea, theanine.

We first examined the distribution of seven major free AAs, urea and ammonium in six different tea plant tissues: the shoot tip including the shoot tip (the apical bud and first leaf) (ST), stem segment 1 (SS1), stem segment 2 (SS2), stem segment 3 (SS3), stem segment (SS4), and the root of 1.5-year-old tea seedlings grown hydroponically in SK medium (Fig. 1A) [26]. Roots had the highest level of Thea (3414 μg/g), ~36-fold higher than that in SS4, and ~7-fold greater than in ST, 8-fold greater than in SS1, 13.8-fold greater than in SS2, and 22.6-fold greater than in SS3. The Arg displayed the highest content in SS1(3546 μg/g), which was more than 10-fold higher than that in roots, and decreased gradually in stems from the top to the lower positions, SS2 (2797 μg/g), SS3 (2422 μg/g), and SS4 (1847 μg/g); but was the lowest content in ST, which was ~20-fold lower than in SS1 (Fig. 1B). By contrast, the Glu content was the highest in ST (1094 μg/g), and decreased gradually in stem segments from the top (971 μg/g in SS1) to the lower positions (638 μg/g in SS4) (Fig. 1B), and was 3-fold higher than in roots. The Gln content was much higher in the root (1003 μg/g), and less abundant in ST and stems, ranging between 200–300 μg/g, resulting from the strong N assimilation activity (Fig. 1B; Dataset S1, see online supplementary material). Urea was only detected in the root (29 μg/g). The NH_4_^+^ content in the root was 54 μg/g, 6-fold higher than in ST, which was changed from 16 μg/g in SS4, 124 μg/g in SS3, 39 μg/g in SS2, to 27 μg/g in SS1, suggesting an active NH_4_^+^ generation and recycling in these stem segments. Correspondingly, the Orn content was higher in roots (42 μg/g), steadily increased from the basal stem SS4 (4 μg/g) to the top positions, SS3 (11 μg/g), SS2 (19 μg/g), to SS1 (22 μg/g), but was not detected in ST. By contrast, Cit (a constituent of the same metabolic cycle) showed higher content in SS1 (36 μg/g), and gradually decreased to 10 μg/g in SS4, and 5 μg/g in roots, indicating that SS1 and roots had opposite metabolic directions for Arg degradation and synthesis, respectively (Fig. 1B; Dataset S1, see online supplementary material).

We further analysed the AA distribution patterns in these tissues of tea plant seedlings fed with 5 mM NH_4_^+^ (Fig. 1B; Dataset S2, see online supplementary material). The changing trends of these eight AAs were very similar to those in non-fertilized tea plant seedlings, but their contents were generally considerably higher upon NH_4_^+^ supply. Indeed, the contents of Thea, Arg, Glu, Gln, Orn, and Cit were significantly higher in all tissues of NH_4_^+^-fed plants than in non-fertilized plants. NH_3_ contents were significantly higher in SS1 and SS2 at 240 h, while Thea, Arg, Gln, NH_4_^+^, Cit, and Orn contents were significantly and differentially increased in SS3, SS4, and roots at 72 or 240 h after feeding. Arg, Urea, and Orn contents were significantly higher in the root at 72 h. The Thea content peaked in the root being considerably lower in stems, but significantly increasing in apical buds and young leaves. By contrast, Arg had the lowest content in roots and ST, Glu was at the lowest level in roots, but Arg and Glu had the highest levels in all stem segments from SS1 to SS4.

A pie chart analysis of six major free AAs, urea and ammonium for their proportions in total NH_2_-containing metabolites of the six tissues (Fig. 1C; Fig. S1, Datasets S1 and S2, see online supplementary material), with the sizes of the pies being proportional to the total AA contents, while the size of the slices represent the proportion of different AAs. From this analysis Arg, Gln, Glu, and Thea appear to be the major components in stems.

### Expression of genes involved in N metabolism in tea root, stem, and leaf

**Figure 2:**
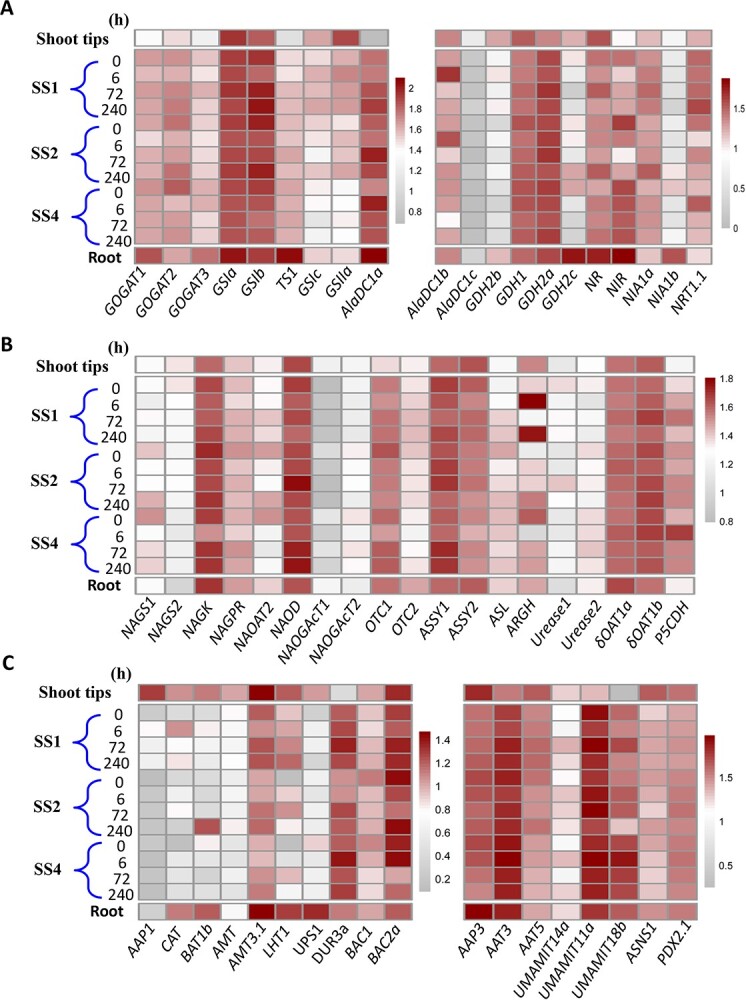
Transcription profiling of nitrogen uptake and assimilation, AA synthesis, transport, and degradation and recycling genes in tea plants. The expression profiles of genes involved in N assimilation and transport (**A**), Arg-Urea synthesis and degradation pathways (**B**), and amino acid transport (**C**) in the root, stem segments (SS), and shoot tip (the apical bud and the first young leaf) of tea plant seedlings fed with 5 mM NH_4_^+^ were obtained with RNA-Seq. Data are from three independent experiments with biological replicates. δOAT, ornithine δ-aminotransferase; AAP, amino acid permease; AlaDC, alanine decarboxylase; AS, arginase; ASL, argininosuccinate lyase; ASSY, argininosuccinate synthase; BAC, Arg/Orn transporter; BAT, bidirectional amino acid transporter; CAT, amino acid transporter; DUR, dural polyamine and urea transporter; GDH, glutamate dehydrogenase; GOGAT, Gln-2OG aminotransferase; GS, glutamine synthetase; LHT, lysine histidine transporter; NAGK, N -acetylglutamate kinase; NAGPR, N-acetylglutamate-5-P reductase; NAGS, N-acetylglutamate synthase; NAOAT, N2-acetylornithine aminotransferase; NAOD, N-acetylornithine deacetylase; NAOGAcT, N-acetylornithine: N-acetylglutamate acetyltransferase; NIR, nitrite reductase; NRT, nitrate transporter; OTC, Orn transcarbamoylase; P5CDH, Δ(1)-pyrroline-5-carboxylate dehydrogenase; PDX2, pyridoxine biosynthesis 2; TSI, theanine synthetase I; UMAMIT, usually multiple acids move in and out transporter; UPS, urea-related transporter.

To better understand whole plant AA metabolism, we next conducted RNA-Seq analyses on the roots, stem segments (SS1, SS2, SS4), and shoot tips. Comparison of all metabolic genes involved in the biosynthesis and conversion of major free AAs revealed that among putative genes involved in Thea biosynthesis pathways, *CsGOGATs* and *CsGSIa*, *CsGSIb*, *CsTS1* were expressed at much higher levels in roots than in ST, consistent with higher Thea contents in roots than in ST. Moreover, the *CsGSIa* transcript was the highest in roots, ~3-fold greater than in SS4, 1.6-fold greater than in ST, with an increasing trend from SS4, SS2, SS1 to ST (Fig. 2A). *CsGSIa* expression patterns were consistent with Thea accumulation patterns in these tissues, so was *CsGSIb* transcript (Fig. 2A). Among four *CsGDH* genes, two (TEA034004 and TEA031206) had higher expression levels in root than in leaf, and the transcript level of *CsGDH* (TEA006665) increased from SS4 to SS2 to SS1, reaching the highest level in ST. Among three *CsAlaDC*s, the expression of one (TEA005658) peaked in the roots. *CsNIR* displayed a higher expression levels in roots and SS4. *CsNRT1.1* (TEA007060) was most highly expressed in SS1, while *CsNR* and *CsNIA1b* transcript levels were highest in roots and *CsNIA1a* displayed higher expression in stems (Fig. 2A; Dataset S3, see online supplementary material). We also analysed genes involved in the Arg biosynthesis pathway from Glu via Orn. The transcript levels of tea N-acetylglutamate synthase 1 (*CsNAGS1*), N-acetylglutamate acetyltransferase 2 (*CsNAOGAcT2*), and N-acetylornithine deacetylase (*CsNAOD*) were higher in SS4 than in roots, N-acetylglutamate kinase (*CsNAGK*), N-acetylglutamate-5-P reductase (*CsNAGPR*), N2-acetylornithine aminotransferase 2 (*CsNAOAT2*) transcript levels were lower in ST than in SS1 and *CsNAOGAcT2* transcript levels were higher in ST than SS1. The *CsNAGS2* transcript showed a slight increase from roots through stems to ST. By contrast, the transcripts of *CsARGH* and urea degradation genes *CsUrease1* displayed higher levels in the stem and shoot tips than other tissues. The pathway for Arg biosynthesis from Orn involves *CsOTC1*, *CsASSY1*, *CsASL*. The transcript levels of these genes were higher in SS4 than in roots, and also higher in SS1 than in ST. *CsδOAT* and *CsP5CDH* involved in the conversion of Orn to Δ1-pyrroline-5-carboxylate (P5C) and P5C to Glu, respectively, were also examined, two *CsδOAT*s, *CsδOAT1a* (TEA033776) displayed a higher expression level in roots, while *CsδOAT1b* (TEA033781) was expressed at higher levels in stems, so was *CsP5CDH* transcript (Fig. 2B; Dataset S3, see online supplementary material).

These gene expression profiles are consistent with AA accumulation patterns in these tissues. Transamination of Glu with oxaloacetate by aspartate aminotransferase (AAT) leads to the formation of aspartate. *CsAAT3* had the highest expression level in SS4 and roots, but this decreased from SS4 up to ST. *CsAAT5* was expressed higher in ST and SS1. *CsASNS1* transcript had the highest level in ST. The Thea hydrolase gene *CsPDX2.1* showed a higher expression level in ST and roots, and displayed a slight decrease from SS4 up to SS1 (Fig. 2B).

Several AA transporter genes showed higher expression levels in the stems and shoot tips, indicating that they likely facilitate AA transport [34, 35]. *CsAAP3* displayed the highest expression levels in the root. More importantly, the Urea-related transporter gene *CsUPS1* displayed the highest expression levels in roots, whereas *CsDUR3a* displayed higher expression in stems, consistent with the highest urea level in roots and the presence of active urea catabolism in these tissues. Two mitochondria Arg/Orn transporter gene *CsBAC1/2* homologs consistently displayed the highest expression levels in the stems, where active Arg-Orn-Cit metabolism is required for the efficient transport of Arg, Orn, and Cit in and out of the chloroplast and mitochondria for conversion into urea, Glu, and other AAs (Fig. 2C; Dataset S3, see online supplementary material). The tea plant homologs of Arabidopsis Arg transporters UmamiT14, 11, and UmamiT18 were also highly expressed in the stem vascular tissues and enhanced upon NH_4_^+^-supply (Fig. 2C; Datasets S3 and S4, see online supplementary material).

### Dissection of genes involved in N uptake and assimilation in roots fed with NH_4_^+^

**Figure 3:**
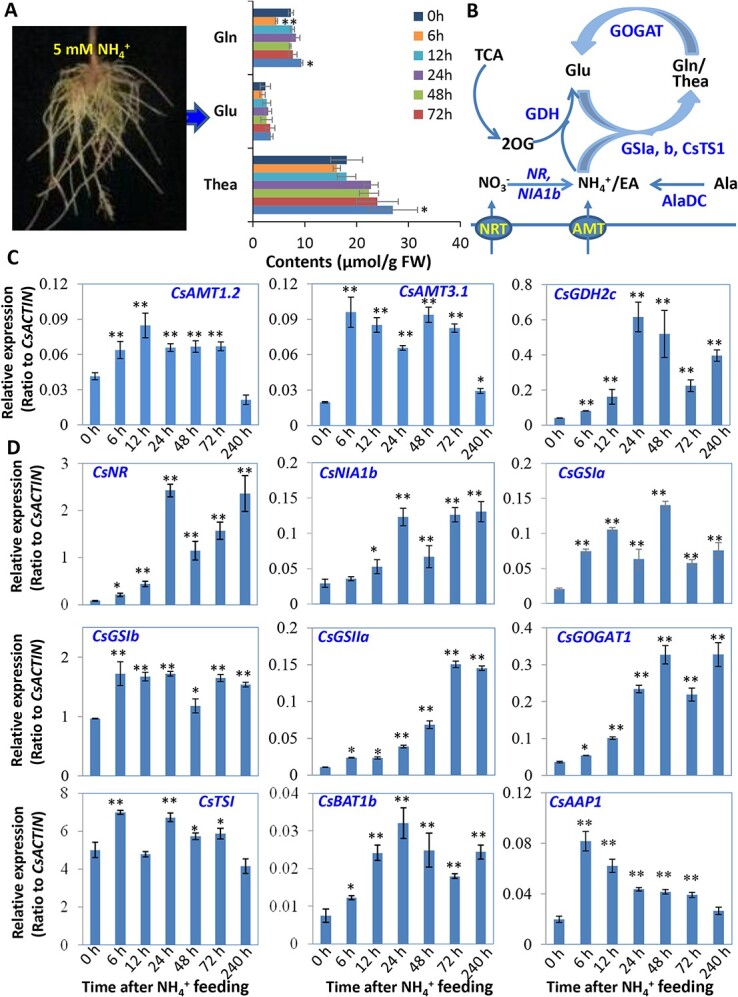
Expression patterns of genes involved in nitrogen uptake and assimilation in tea roots. (**A**) Photo for tea seedling roots fed with 5 mM NH_4_^+^ (left panel) and the major amino acid profiles (right panel). (**B**) The N uptake and assimilation for Glu, Gln, and Thea biosynthesis. (**C**–**D**) qRT-PCR analysis of expression patterns for genes involved in NH_4_^+^ uptake and assimilation, and biosynthesis of Gln and Thea. Data are from at least three independent experiments with biological replicates and are expressed as means ± SD. Differences between treatment and control are considered significant when ^**^P < 0.01 and ^*^P < 0.05. AAP, amino acid permease; AMT, ammonium transporters; BAT, bidirectional amino acid transporter; GDH, glutamate dehydrogenase; GOGAT, Gln-2OG aminotransferase; GS, glutamine synthetase; NIA, nitrate reductase; NR, nitrate reductase; TSI, theanine synthetase I.

As the contents of Thea, Gln, and Glu in tea roots increased in response to 5 mM NH_4_^+^supply (Fig 3A), we further analysed early N responsive genes under supply of 5 mM NH_4_^+^ [1]; Ruan et al., 2010; [36]; Zhang *et al.*, 2022. *CsAMT1.2* and *CsAMT3.1* were up-regulated, peaking at 12 h and 6 h, followed by recovery to basal levels. Our results suggested that NH_4_^+^ was assimilated through GS/GOGAT cycle and GDH enzymes (Fig. 3B), with *CsGDH2c* being up-regulated, peaking at 24 h; *CsNR*, *CsNIA1b* and *CsGOGAT* and *CsGSIa* expression fluctuated at higher levels from 12 h to 240 h and NH_4_^+^-supply also up-regulating *CsTS1*, *CsGSIb*, and *CsGSIIa,* whose expression peaked at 6 h, 24 h, and 72 h, respectively (Fig. 3C). Both AA transporter *CsBAT1b* and *CsAAP1* were up-regulated with expression peaking at 24 h and 6 h, respectively (Fig. 3D).

### Expression patterns of genes involved in N assimilation and metabolism in roots

**Figure 4:**
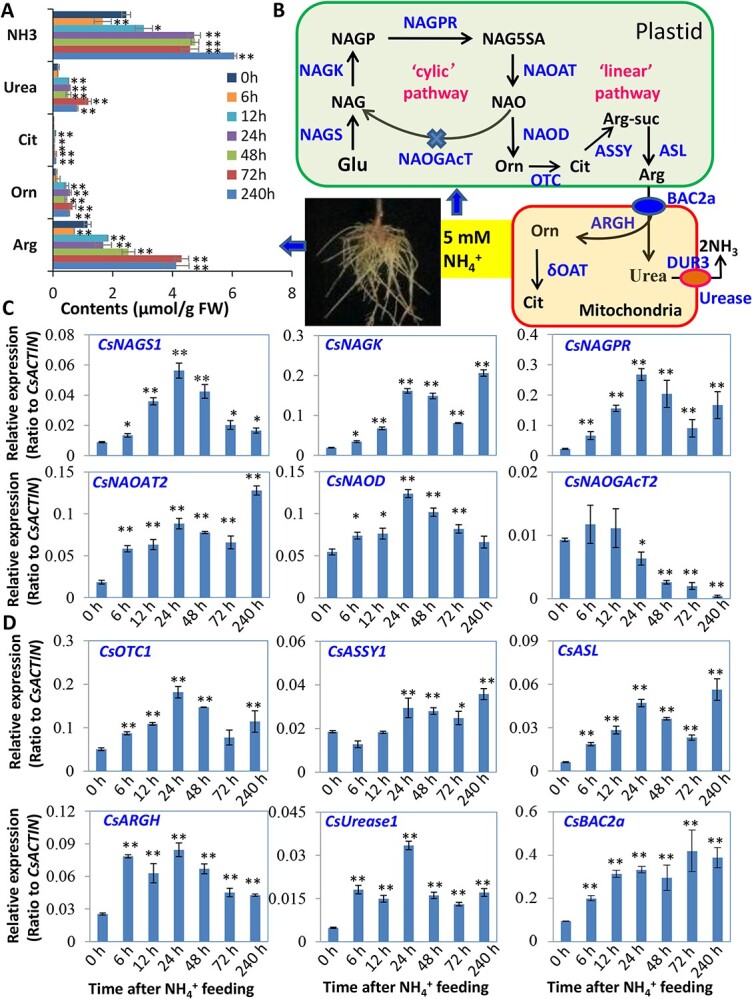
Profiles of metabolites and genes involved in Arg-Urea biosynthesis and transport in tea roots upon NH_4_^+^ supply. (**A**) The profiles for major NH_2_-containing metabolites involved in Arg-Urea biosynthesis pathway in tea roots under NH_4_^+^ supply. (**B**) Genes involved in Glu-Orn-Cit-Arg-Ura metabolic pathway in the plastid and Arg degradation in the mitochondrion. (**C**–**D**) qRT-PCR examination of related gene expression in roots in response to NH_4_^+^ supply. Data are from at least three independent experiments with biological replicates and are expressed as means ± SD. Differences between treatment and control are considered significant when ^**^P < 0.01 and ^*^P < 0.05. δOAT, ornithine δ-aminotransferase; ARGH, arginine hydrolase; ASL, argininosuccinate lyase; ASSY, argininosuccinate synthase; BAC2a, Arg/Orn transporter; DUR3, Urea transporter; NAGK, N -acetylglutamate kinase; NAGPR, N-acetylglutamate-5-P reductase; NAGS, N-acetylglutamate synthase; NAOAT, N2-acetylornithine aminotransferase; NAOD, N-acetylornithine deacetylase; NAOGAcT, N-acetylornithine: N-acetylglutamate acetyltransferase; OTC, Orn transcarbamoylase.

Urea is a N storage metabolite derived either from root N uptake or from catabolism of Arg by CsARGH [3]. The Orn, Arg, and urea contents increased in response to 5 mM NH_4_^+^-supply (Fig. 4A). In three routes towards Arg biosynthesis, Orn is synthesized from Glu either in a ‘cyclic’ or a ‘linear’ pathway, followed by Arg degradation to urea (Fig. 4A and B). qRT-PCR validation of the transcriptome data on the Orn-Arg-Urea pathway genes tea roots in response to 5 mM NH_4_^+^-supply was conducted (Fig. 4C; Fig. S3, see online supplementary material). *CsNAGS1* expression was up-regulated pronouncedly at 24 h, followed by a decrease*.* The rate-limiting gene *CsNAGK* was significantly up-regulated, peaking at 240 h while the *CsNAGPR* transcript displayed a pronounced peak at 24 h. *CsNAOAT2* and *CsASL* transcripts displayed a significant increase at 240 h in a similar pattern following N-feeding with *CsNAGK,* while *CsNAOD* and *CsOTC1* transcripts showed a slight increase at 24 h, followed by a steady decrease (Fig. 4C)*. CsASSY1* showed a slight decrease, followed by recovery at 12 h and an increase at 24 h (Fig. 4C)*.* The *CsARGH* transcript increased at 24 h, followed by a decrease, while the *CsUrease1* transcript displayed a significant increase at 24 h. The transcripts for *CsBAC2a* constantly increased under NH_4_^+^-supply (Fig. 4C). In the ‘cyclic’ pathway, *CsNAOGAcT2* only showed a slight increase at 6 h and 12 h, followed by a rapid decrease (Fig. 4C), perhaps suggesting a dominant metabolic flux from Glu to Orn through the linear pathway (Fig. 4B).

**Figure 5:**
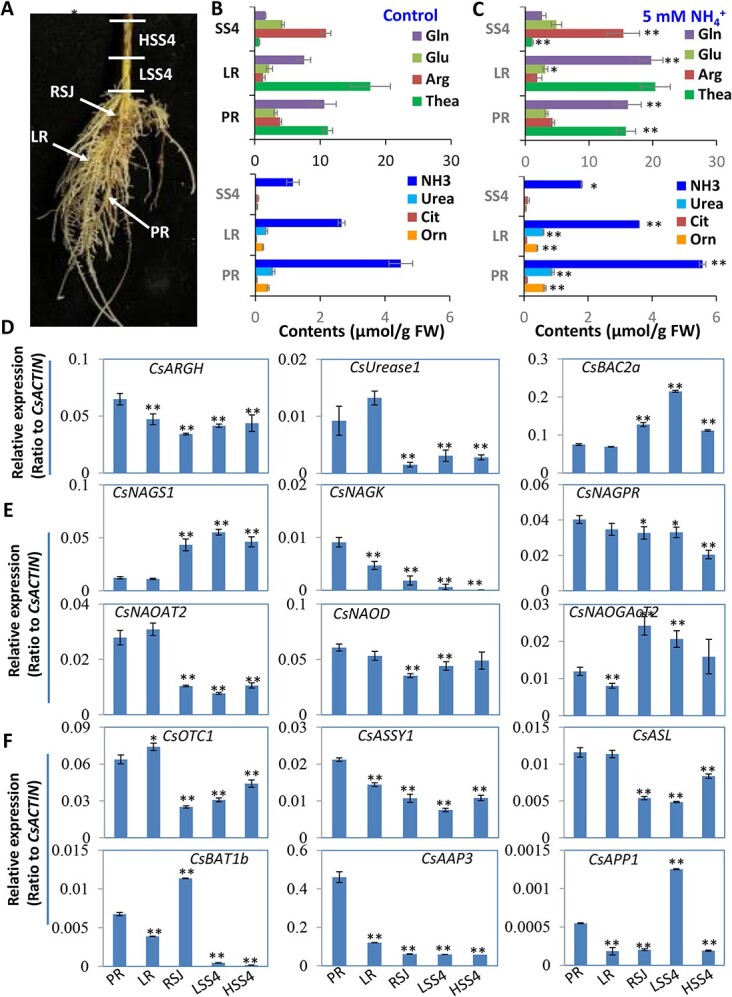
Dissection of Arg metabolism and transport genes in different parts of tea roots. (**A**) The tea root image of a two-year old tea seedling grown hydroponically in SK media and the dissection into different parts for analysis of AAs and metabolic genes. HSS4, higher stem segment 4; LR, lateral roots; LSS4, lower stem segment 4; PR, primary roots; RSJ, root-shoot joint. (**B**, **C**) Profiles of the major AAs and related NH_2_-containing metabolites in dissected parts of tea roots. (**D**–**F**) qRT-PCR validation of gene expression patterns for Arg synthesis (**D**, **E**), and amino acid transport (**F**) in dissected parts of tea roots. Data are from at least three independent experiments with biological replicates and are expressed as means ± SD. Differences between treatment and control are considered significant when ^**^P < 0.01 and ^*^P < 0.05. AAP, amino acid permease; ASL, argininosuccinate lyase; ASSY, argininosuccinate synthase; BAT, bidirectional amino acid transporter; NAGK, N -acetylglutamate kinase; NAGPR, N-acetylglutamate-5-P reductase; NAGS, N-acetylglutamate synthase; NAOAT, N2-acetylornithine aminotransferase; NAOD, N-acetylornithine deacetylase; NAOGAcT, N-acetylornithine: N-acetylglutamate acetyltransferase; OTC, orn transcarbamoylase.

### Dissection of AA metabolism in different parts of the tea root

To understand the detailed changes in AA metabolism in the root and stem, the root and basal stem were dissected into five segments—primary roots (PR), lateral roots (LR), root-shoot joints (RSJ), and lower stem segment 4 (LSS4) and the higher stem segment 4 (HSS4)—to decipher Arg synthesis pathway genes (Fig. 5A and B). AA profiles in these tissues showed that Thea contents were highest in LR and lowest in SS4; Arg content was the highest in SS4, but the lowest in LR. Thea content was ~13-fold higher than Arg in LR，but the Arg content was ~15 fold higher than Thea in SS4. The NH_3_, Orn, and Urea contents decreased from PR, LR to SS4 (Fig. 5B). All of these AA contents increased upon 5 mM NH_4_^+^-supply (Fig. 5C).

The transcripts of *CsNAGS1* were at their lowest level at PR and LR, but at higher levels in RSJ, LST4, and HST4, whereas the rate-limiting gene *CsNAGK* transcript displayed an opposite pattern (Fig. 5D and E). *CsAGRH*, *CsNAGPR,* and *CsNAOD* transcripts were also higher in PR and LR, then decreased in RSJ, LSS4, and HSS4; and transcripts of *CsUrease1*, *CsNAOAT2, CsOTC1, CsNAGK, CsASSY1,* and *CsASL* were also higher in PR and LR but lower in RSJ, LSS4, and HSS4 (Fig. 5D–F). Meanwhile *CsNAGAcT* was observed to be lowly expressed in LR but its expression was higher in RSJ (Fig. 5E and F). These data suggest that primary roots (PR) and lateral roots (LR) had predominant Arg and Urea synthesis activity via the Glu-Orn-Cit-Arg-Urea pathway. By contrast, young leaves prominently displayed Arg-Urea degradation activity via Arg-Urea-Orn/NH_3_-Glu for AA recycling and NH_3_ re-assimilation. Thus, Arg and Urea were actively biosynthesized in PR and LR, transported to root-shoot joints (RSJ) for root-shoot long-distance transport to upper stems, then Arg was efficiently down-loaded to leaves where it was degraded. The young leaves had the strongest Arg catabolism and Thea and Gln biosynthesis. The expression level of the ‘cyclic pathway’ gene *CsNAOGAcT* was higher in RSJ, LSS4, and HSS4 than in PR and LR (Fig. 5E). Furthermore, the transcript level of the AA transporter *CsBAT1b* gene was the highest in RSJ, higher in PR, but the lowest in LSS4 and HSS4. *CsAAP3* also had the highest transcription level in PR, significantly decreased in LR, RSJ, LSS4, and HSS4 (Fig. 5F). *CsBAC2a* and *CsAAP1* also displayed the highest levels of transcription in LSS4, and lower levels in RSJ, PR, and LR (Fig. 5D and F). These are consistent with their tissue-specific expression patterns (Dataset S5, see online supplementary material).

### Arg, Glu, Gln, and Thea are the major AAs in xylem sap for N transport in tea stem

**Figure 6:**
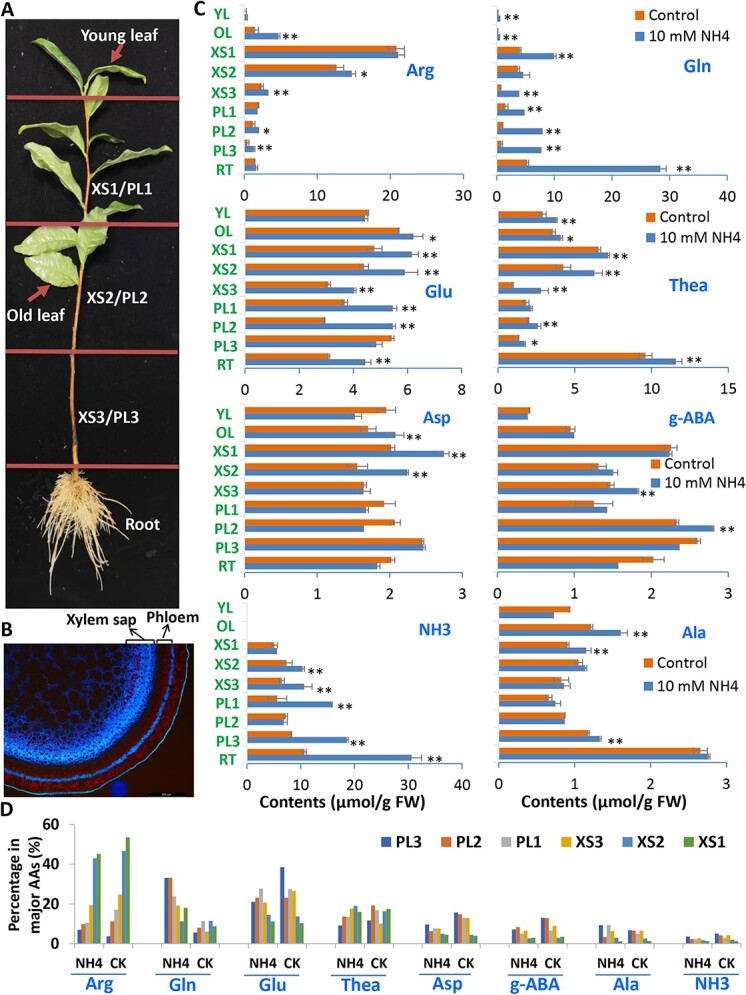
Major AAs and NH_2_-containing metabolites in xylem sap of tea stem fed or non-fed with NH_4_^+^**.** (**A**) Photo indicating the different stem fragments for xylem sap1, 2, 3 (XS1, XS2, and XS3), phloem 1, 2, 3 (PL1, PL2, and PL3), and young leaf and root. The contents of major amino acids and NH_2_-containing metabolites in xylem sap of tea stem fed or non-fed with NH_4_^+^. (**B**) Image of tea stem cross-section under UV light. Blue fluorescence signals indicate vascular tissues of lignin polymers. (**C**) Profiles of major AAs and NH_3_ in tea xylem sap, phloem, and leaf and root tissues grown in SK media supplied with NH_4_^+^ (NH_4_) and without supply (control). (**D**) Percentage for each major AA in total AA contents of xylem sap, phloem, and whole root under NH_4_^+^ supplement (NH_4_) or without supplement (CK). Data are expressed as means ± SD from at least three independent experiments with triplicate repeats. Differences between treatment and control are considered significant when ^**^P < 0.01 and ^*^P < 0.05.

To further confirm that Arg, Glu, and other AAs as AA transport forms in tea stems from the root to the shoot tip, we profiled AAs in the xylem and phloem (Fig. 6A and B; Dataset S6, see online supplementary material). Xylem sap entering the shoot contained large amounts of AAs, 20–45% (from lower to upper stem’s xylem sap) of which was Arg (representing the major xylem AA), followed by Glu (10%–26%), Thea (~11%–19%), and Gln (~6%–19%), Asp (~4–12%), g-ABA (~2–9%), NH3 (~1–6%), and Ala (~1–4%) (Fig. 6C and D). Arg contents increased from lower xylem sap XS3 to upper positions XS2 and XS1, and also were lower (~45%) in 5 mM NH_4_^+^ supply than in control (~53%). Glu in xylem sap accounted for 10–26% showing the opposite trend, higher in low XS3 and lower in upper xylem saps XS2 and XS1. Thea in xylem saps represented about 10–19% of total AAs. NH_4_^+^-supply significantly enhanced Gln accumulation in xylem sap and phloem. Glu and Gln were found as the dominant AAs in tea stem phloem, which were also dramatically affected by NH_4_^+^-supply, as compared with control (Fig. 6C). 5 mM NH_4_^+^-supply reduced the proportion of Glu in xylem sap, but increased Gln proportion to the highest level (23–33%) (Fig. 6D; Dataset S6, see online supplementary material).

The expression of several AA transporter genes supported Arg biosynthesis and degradation in response to NH_4_^+^-supply (Fig. S4, see online supplementary material). *CsCAT* and *CsLHT1* transcripts increased significantly at 72 h or 240 h in SS4 and SS2, and *CsCAT* was also up-regulated at 72 h in SS1. AtBAT1 can import Ala and Arg and export Glu and Lys [13]. *CsBAT1b* was most highly expressed in RSJ and PR. *CsBAT1b* was up-regulated by NH_4_^+^supply with a significant increase at 72 h in SS4, SS2, and SS1 (Fig. S4, see online supplementary material). Two Arg/Orn transporter genes *CsBAC1/2* exhibited their highest expression levels in stems (Fig. S4, see online supplementary material). The changes were also detected in *CsAAP1* and *CsAAP3* expression levels in different stems (Fig. S4, see online supplementary material). *CsARGH* was also significantly induced in different stem segments by NH_4_^+^-supply, in a similar pattern to that observed by *CsBAT1*. The *CsUrease1* transcript also was significantly increased at 240 h stems (Fig. S4, see online supplementary material), all suggesting an enhanced Urea metabolism and AA transport processes in response to NH_4_^+^-supply.

### Degradation of Arg and Gln/Thea synthesis in tea plant shoot tips and young leaves

**Figure 7:**
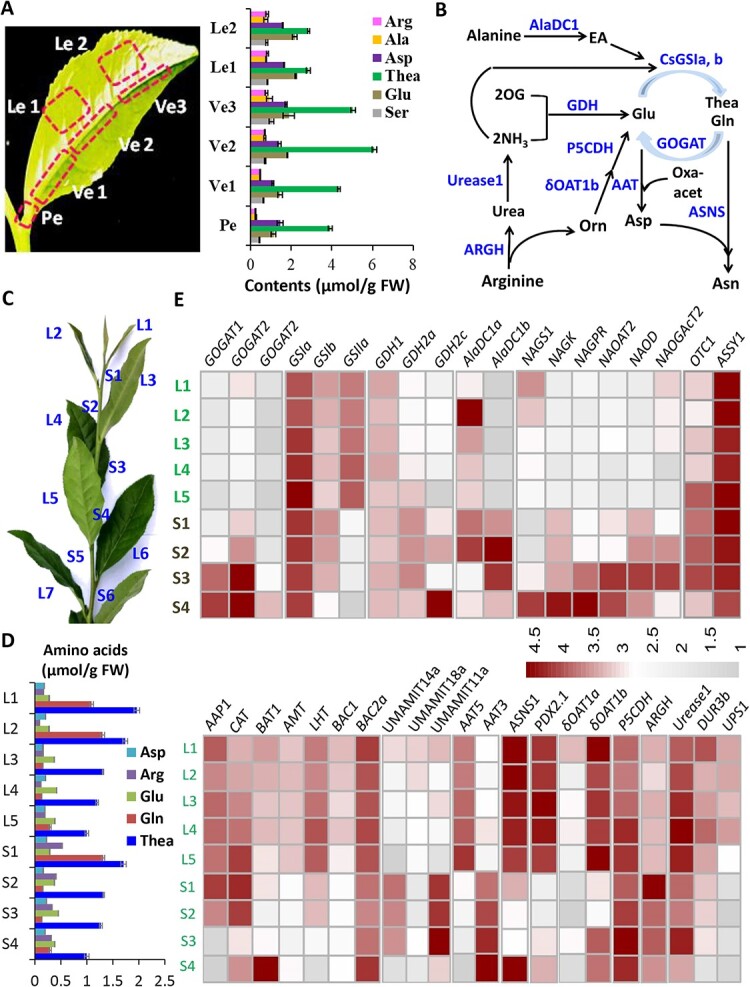
Expression patterns of genes involved in AA metabolism and transport in tea shoot tips. (**A**) Dissection of the first tea leaf for AA distribution. Left panel: the different parts of the first leaf were indicated: Pe, Petiole; Ve-1, Vein 1; Ve-2, Vein 2; Ve-3, Vein 3; Le-1, leaf area 1; Le-2, Leaf area 2; Right panel: profiles of major AAs in these leaf parts. (**B**) The metabolic pathways and metabolic genes involved in biosynthesis of the major AAs. (**C**) The leaves and internodes in the top shoot of an eight-year-old tea tree. (**D**) The AA profiles in leaves and internodes of tea top shoots. (**E**) Heat map analysis of the expression patterns of N assimilation and Thea/Gln synthesis genes, Arg-Urea synthesis/degradation and related metabolic genes, and AA transporter genes in various leaves and stem internodes, The AA metabolic genes and transporter genes were retrieved from previous RNA-Seq data. Data are expressed as means ± SD from at least three independent experiments with triplicate repeats. Differences between treatment and control are considered significant when ^**^P < 0.01 and ^*^P < 0.05. δOAT, ornithine δ-aminotransferase; AAP, amino acid permease; AlaDC, alanine decarboxylase; AMT, ammonium transporters; ASSY, argininosuccinate synthase; BAC, Arg/Orn transporter; BAT, bidirectional amino acid transporter; CAT, amino acid transporter; DUR, dural polyamine and urea transporter; GDH, glutamate dehydrogenase; GOGAT, Gln-2OG aminotransferase; GS, glutamine synthetase; LHT, lysine histidine transporter; NAGK, N -acetylglutamate kinase; NAGPR, N-acetylglutamate-5-P reductase; NAGS, N-acetylglutamate synthase; NAOAT, N2-acetylornithine aminotransferase; NAOD, N-acetylornithine deacetylase; NAOGAcT, N-acetylornithine: N-acetylglutamate acetyltransferase; OTC, Orn transcarbamoylase; P5CDH, Δ(1)-pyrroline-5-carboxylate dehydrogenase; UPS, urea-related transporter.

Further dissection of the first tea leaf into petiole (Pe), vein 1 (Ve1), vein 2 (Ve2), vein 3 (Ve3), leaf area 1 (Le1), Leaf area 2 (Le2) (Fig. 7A) for AA profiles revealed that Thea was the major AA and increased from Pe, Ve1, to Ve2; Arg content was the lowest in Pe, then increased from Pe, Ve1, Ve2, Ve3, to Le1 (Fig. 7A). Whereas Glu, Asp, and Ala contents were dramatically reduced compared with these in SS1 (Fig. 7A; Dataset S7, see online supplementary material). Thea was mainly accumulated in vascular parts, such as midvein and petiole and veins, but lower in leaf areas, which is consistent with a recent report (Fig. 7A) [37]. Arg, Glu, and Asp contents were higher in leaf areas and older veins, indicating the AA metabolism in young leaves was different from stems and roots (Fig. 7A and B).

To understand the changes in AA metabolism in stems and shoot tips, we next dissected the shoot of tea plants of 8-year-old tea plants and profiled the AAs in nine tea plant tissues: the first leaf from the top (L1), the second leaf (L2), the third leaf (L3), the fourth leaf (L4), the fifth leaf (L5), and the first (top) internode between the first leaf and the second leaf (S1), and the second internode between the second leaf and the third leaf (S2), and so on for S3 and S4 (Fig. 7C) [38]. Thea and Gln contents displayed a continuous increase from S4 up to S1 and from L4 up to L1 (Fig. 7D). The Arg content continuously increased from S4 up to S1. The Glu and Asp content had a little change in these tissues (Fig. 7D; Dataset S8, see online supplementary material).

Transcriptome analysis was conducted on these leaves (L1–L5) and internodes (S1–S4) (Fig. 7E) [38]. Transcripts of three *CsGOGATs* increased from S1 to S4, particularly *CsGOGAT2* increased drastically. *CsUrease1, CsDUR3b, CsGSIa*, *CsGSIb*, and *CsGSIIa* transcripts increased from S4 to S1(Dataset S9, see online supplementary material). Meanwhile, *CsAlaDC1a* and *CsGSIb* were highly expressed in shoot tips with the highest levels in young leaves, and *CsAlaDC1b* was highly expressed in stems, increasing from S4 up to S1, *CsAlaDC1a* and *CsGSIa* transcripts increased to higher levels in young leaves, in consistence with Thea accumulation patterns. *CsGDH1*, *CsGDH2a*, and *CsGDH2c* had higher expression levels in stems than in leaves, together with higher transcript levels of *CsNAGK*, *CsNAGPR*, *CsNAOAT*, *CsNAOD* involved in Glu-Orn-Arg biosynthesis in stems than in young leaves, suggesting that active Arg conversion in Glu-Arg-Orn-Ura-Glu cycle in stems. These gene expression and AA profiles were consistent with higher Arg accumulation level in stems and active Arg degradation in shoot tips and young leaves (Fig. 7E). Consistently, *CsδOAT1a* and *CsδOAT1b* had higher transcript levels in L1 and *CsP5CDH* showed an increased transcript from young to old stems and leaves (Fig. 7E, Fig. S5; Dataset S9, see online supplementary material). The Urea transporter *CsUPS1* was more highly expressed in leaves than in stems. The *CsAAP1* transcript increased from L2 to L4, with the highest level in S1. *CsCAT* transcript increased from L1 to L5. *CsBAT1b* transcripts decreased from L1 to L3, but were higher in S4, which contained a larger amount of Arg than the roots (Fig. 7F). *CsAMT*, *CsLHT1*, and *CsBAC1* displayed higher expression levels in leaves than in stems. *CsBAC2a* transcripts decreased from L1 to L3, but were also higher in S4 than in other tissues. The expression patterns of these transporter genes were basically consistent with Arg metabolism patterns in stems and leaves tissues. The transcripts of two genes encoding the putative Arg/Orn/Cit transporters, *CsUMAMIT14a* and *CsUMAMIT18a*, decreased from L1 to L5 and *CsUMAMIT14a* transcripts decreased from S1 to S4. *CsUMAMIT11a* showed higher expression level in S3, S1, and S2 than in leaves (Fig. 7G; Dataset S9, see online supplementary material). These expression patterns were consistent with active Agr/Orn metabolism patterns in stems and young leaf tissues, indicating an active NH_3_ recycling into Glu. Arg/Orn transport and metabolism patterns were also similar to Thea accumulation patterns, which increased from lower stem S4 up to higher stem S1 position, and older leaf L5 up to young leaf L1, indicating they might be correlated by inter-conversions in young stems and leaves, where indeed Thea synthesis occurred. Arg levels were 5–8 times lower in leaves and roots than in stems, consistent with the transcriptome data about related metabolic gene expression, further supporting that Arg is the major transport AA in tea plants.

### Essential roles of CsGSIa in root N assimilation and shoot AA recycling

**Figure 8:**
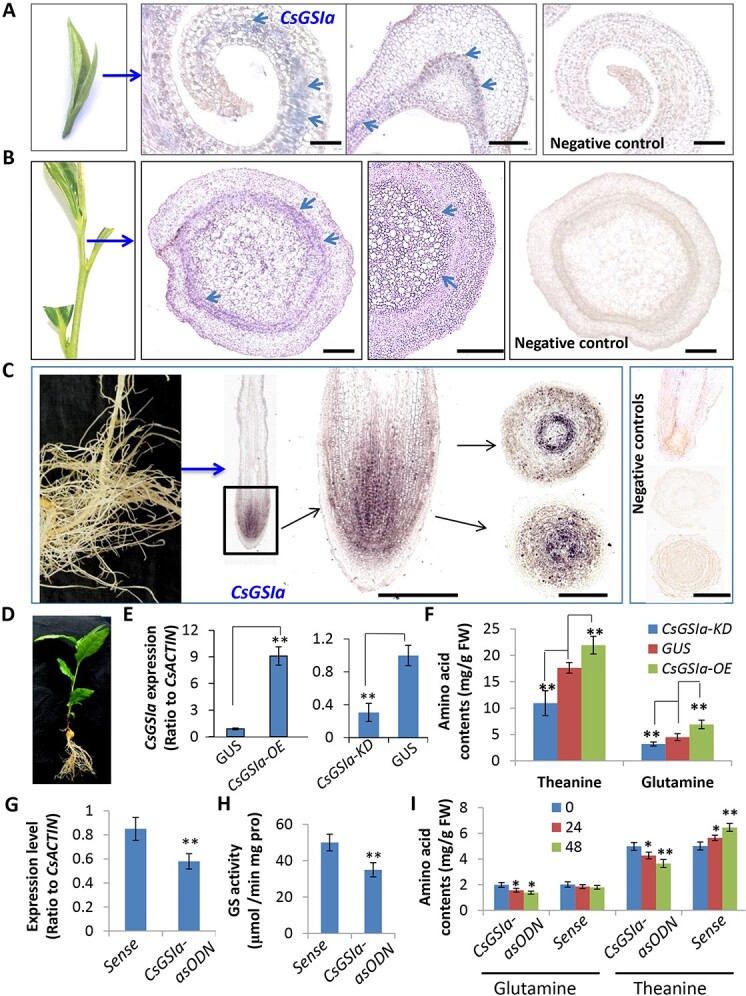
Characterization of *CsGSIa* in tea plants for Thea and Gln biosynthesis and transport. (**A**) *In situ* hybridization for *CsGSIa* transcripts in an apical bud. Blue positive signals were seen in the mesophyll cells of the upward side (adaxial side) of young leaf blade. Bar = 300 μM. (**B**) *CsGSIa* transcripts in the stem. The positive signals were primarily in the endodermis zone of stems, as well as vascular tissues. Bar = 200 μM (**C**) *CsGSIa* transcripts in the root tips. The signals were primarily in the meristem zone of root tips, as well as vascular tissues in the elongation zone. Bar = 200 μM The negative controls without using probe showed no transcript signal detected *in situ* hybridization on apical bud, stem and root tip samples. (**D**–**E**) The representative photo for the transgenic chimerical tea seedling (**D**) and qRT-PCR analysis of transgene in overexpression (*CsGSIa-OE*) and RNAi knockdown (*CsGSIa-KD*) tea hairy roots (**E**). (**F**) Theanine contents in the transgenic hairy roots, *CsGSIa-OE* and *CsGSIa-KD*, as compared with *GUS* control hairy roots. (**G**, **H**) The asODN inhibition of *CsGSIa* expression and the reduced GS activity in the shoot tips (apical buds and young leaves). (**I**) Theanine and glutamine contents in the asODN-treated shoots as compared with control sense ODN. Data are expressed as means ± SD from at least three independent experiments with triplicate repeats. Differences between treatment and control are considered significant when ^**^P < 0.01 and ^*^P < 0.05.

While GS has been shown to participate in N assimilation, translocation, and recycling in model plants [39, 40], and heterologous expression of CsGS1a in tobacco showed a role in theanine synthesis [41], genetic evidence and details of CsGSs in AA metabolism, translocation, and recycling in tea plants are still lacking. To better understand the molecular aspects of root N assimilation, AA long-distance transport, and Arg recycling in shoot tips, we further characterized one of tea major GSs, CsGSIa. *In situ* hybridization showed that *CsGSIa* transcripts were highly accumulated in both roots, stems, and the apical bud and young leaf of the shoot tip (Fig. 8A–C). *CsGSIa* transcripts could be observed in the mesophyll cells in the adaxial side of apical buds and vein vascular tissues of the first leaf, endodermis zone as well as S1 vascular tissues, as well as the meristem zone and vascular tissues of the root. Thus, CsGSIa may play key roles in root N assimilation, AA transport and recycling of NH_3_ generated from Arg-Urea degradation in shoot tips (Fig. 8). We used the RNA interference (RNAi) technique to knock down *CsGSIa* transcription (*CsGSIa-KD*) and 35S-driven overexpression (*CsGSIa-OE*) in tea seedling roots to verify the roles of *CsGSIa*, with rhizobia *Agrobacterium rhizobia*-mediated hairy root transformation [42]. From transgenic hairy root lines of *CsGSIa-KD* and *CsGSIa-OE,* respectively, we detected the reduced and enhanced transcript levels of transgene *CsGSIa* (Fig. 8D and E). Analyses of Thea and Gln contents in these transgenic lines showed that *CsGSIa* RNAi transgenic hairy root lines had reduced Gln and Thea levels, and *CsGSIa-OE* lines with enhanced Gln and Thea contents, as compared with *GUS* control lines (Fig. 8F). Therefore, CsGS1a had critical roles in root N assimilation for Gln and Thea biosynthesis, similar with CsTSI [26, 29].

An antisense oligodeoxynucleotide (asODN)-mediated repression strategy was also used to knock down *CsGSIa* transcript in tea shoot tips (Fig. 8G). Similar to previously reported asODN-mediated gene repression [38], the reduced *CsGSIa* transcript levels in asODN-treated apical buds were observed (Fig. 8G). Following the repressed *CsGSIa* transcription and GS activity (Fig. 8H), apical buds also displayed reduced Gln and Thea contents (Fig. 8I). These data suggested that CsGSIa were responsible for both Gln and Thea biosynthesis during Arg-Urea degradation and AA recycling in ST tissues.

## Discussion

While both Glu and Thea are believed to be generated by TSI and GSI during N assimilation and NH_4_ metabolism, why the level of Thea are usually higher than that of Gln in tea roots and leaves has still puzzled plant researchers [20, 29]. N fertilizer application has been reported to increase Thea and AA contents in tea plant leaves [17, 26], and it is known that Thea can be transported from roots to shoot tips [32]. However, more recent studies also suggested that substantial amounts of Thea in young tea leaves can also be synthesized *de novo* in local shoot tips [29, 30]. As such, a finer analysis of the biosynthesis and transport of Thea is required in tea plants despite the fact that several non-specific AA transporters are well characterized [32, 33, 36]. We dissected whole tea plants to analyse the dynamic profiles of major free AAs, their metabolic pathways and transport mechanisms in different parts of tea plants. We found that N assimilation and AA metabolism in roots were dominated by the Glu-Gln/Thea-Orn-Arg pathway, while the long-distance root-leaf transport of N occurs mainly in the form of Arg, Glu, Gln, and Thea. Moreover, an active Arg degradation and AA recycling are found in young tea leaves, via the Arg-Urea/Orn-NH_3_-Glu pathway. These spatial dynamics of AA metabolic processes critically involve CsGSIa as a key metabolic enzyme that may also facilitate AA recycling and transport. This finding lays an important foundation for future in-depth understanding of Thea biosynthesis and regulation in tea plants, which we believe will facilitate the improvement of Thea production in tea plants by cultivation and tea variety breeding renovation.

### Distinct metabolism and accumulation patterns of major AAs in various tea tissues

We showed that the most significant feature in tea plant N metabolism is the high levels of Thea and Gln in roots and Arg and Glu in stems, as well as efficient conversions between Thea, Gln, Arg, and Glu in tea root, stem, and leaf. In tea roots fed with 5 mM NH_4_^+^, Thea content was about 30% higher，while Glu content was 1.4-fold, Gln content was 1.2-fold, and Arg content was ~3.5-fold higher than those in control (at 0 h time point of treatment). Meanwhile, Urea, Cit, and Orn contents were more than 4–5-fold higher, while the NH3 content was more than 2-fold higher than those in control (at 0 h time point of treatment). Thus, strong N assimilation/NH_4_^+^ detoxification and Arg-Urea biosynthesis activity took place in tea roots fed with 5 mM NH_4_^+^. However, the opposite directionality of this metabolic pathway dominated in young leaves, where Arg and Gln contents significantly decreased, but Glu, Orn, Cit, Urea, and NH_3_, and Thea contents increased, indicating Arg-Urea degradation, NH_3_ re-assimilation, and AA recycling activities in young leaves. The transcriptome data revealed the increased expression levels of Glu-Orn-Cit-Arg pathway genes in roots in line with the increased Arg levels in RSJ and SS4. By contrast, the increased expression levels of genes involved in Arg-Orn/Urea degradation pathway, together with the drastic drop in Arg content increased Glu, Thea, and Gln contents in young leaves, strongly indicate active Arg degradation and AA recycling. The *in-situ* hybridization data showed that *CsGSIa* transcripts presented in root epidermal and vascular, stem vascular, and leaf vein cells strongly support the critical roles of *CsGSIa* in Thea and Gln synthesis, transport, and recycling (Fig. 8).

**Figure 9:**
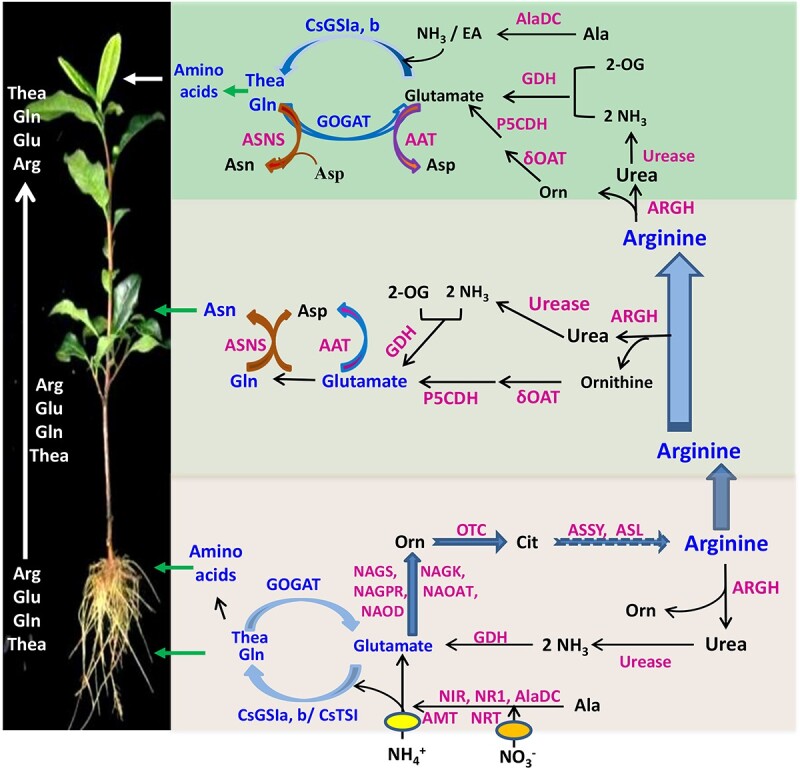
A working model for N assimilation, AA synthesis, transport, and degradation/recycling in tea plants.

In young tea stems, Arg dominates the free AA levels in the xylem sap, together with Glu, Gln, and Thea; the dynamic changes of their concentrations in different stem segment positions imply their roles in root-to-leaf long distance transport via vascular tissues. Arg could be loaded into phloem and xylem sap for long-distance transport, and then downloaded from xylem sap, entering either axillary branches or young leaves for active conversions into other amino acids for supporting plant growth. Consistently, in NH_4_^+^-supplied tea plant seedlings, the contents of AAs including Thea, Gln, Glu, and Arg increased to strikingly higher levels compared with NO_3_^−^ and NO-supplied tea plants [17, 18, 43, 44]. Our profiles of major AAs in tea xylem sap were slightly different from those of Japan tea trees, in which Gln (49%), Thea, Arg, Asp, Glu were the major AAs [43]. This may indicate natural variations in AA contents in different tea cultivars under various physiological and environment conditions [45]. Some woody plants use Arg as a major AA form in root-shoot transport via xylem sap [3, 46]. Despite the complex free AA metabolism in tea plants, Arg, Gln, Glu, and Thea were the major AAs in xylem sap ([43] and this study). Dissections of AA levels and gene expression profiles in tea roots, stem segments, xylem sap, and young leaves clearly supported the distinctive metabolism features in different organs of tea plants. Several lines of evidence show that besides cytosolic GS/GOGAT, cytosolic asparagine (Asp) synthetases (ASNSs) are also responsible for NH_3_ assimilation into AAs [3, 47]. However, neither of two *ASNS* genes were up-regulated by NH_4_^+^-supply, nor were contents of Asp or Asn increased, indicating that ASNSs are not essentially involved in N assimilation (Dataset S4, see online supplementary material). However, in stems and shoot tips, high levels of Asp were observed, and both *CsASNS* and *CsAATs* were highly expressed (Fig. 7G; Dataset S1, see online supplementary material), indicating that these genes also participate in AA transport in stems and recycling in shoot tips. In NH4 + -supplied tea plants, Gln and Arg were the most significantly increased AAs, followed by Glu and Thea (Fig. 1B).

### A‌A transporters facilitate N translocation, utilization, and recycling

As Arg, Glu, Gln, and Thea are the major AAs for the long-distance root-shoot transport in tea seedlings, various amino acid transporter genes were found expressed in related tissues. AtAAP3 has a high affinity to basic AAs [48, 49], and rice OsAAP3 being expressed in the vasculature may function in AA exchange between the xylem and phloem Lu *et al.*, 2018 [35]. Six CsAAPs were shown to transport Gln, Glu, Aspartate (Asp), and γ-aminobutyric acid (g-ABA) in tea roots and responded to N-supply [32, 50]. *CsAAP1* was highly expressed in young tea leaves and stems and *CsAAP3* was highly expressed in roots and young tea leaves, but expressed at a lower level in stems, indicating their roles in facilitating the transport of these major AAs in tea plants for various purposes. The increasing *CsAAP1 and CsAAP3* transcripts in stems from lower to top positions were in a similar pattern with Arg and Glu contents (Fig. S4, see online supplementary material). The Ala/Arg importer and Glu/Lys exporter homolog *CsBAT1b* was expressed to the highest level in RST (Fig. 5F) and significantly up-regulated by NH_4_^+^-supply, indicating a putative role in the Glu and Arg synthesis and transport in RSJ and SS4 and stems. The mitochondrial Arg transporter homolog genes, such as *CsBAC*s or *CsBAT1/3*, were highly expressed in stems (Fig. 7G) and could transport base AAs, such as Arg and Orn, in and out of the mitochondria for degradation by CsARGH. The higher levels of Cit and *CsUPS1* and *CsDUR3b* expression in upper stems and young leaves also implied an active Orn-Cit shuttle to transport Urea and NH_4_^+^ [46]. Two major LHT1 may facilitate root uptake and supply leaf mesophyll with xylem-derived amino acids [14]. The efficient export of Orn by mBAC from the mitochondrion to the cytosol is important for cytosol Orn-Arg conversion in yeast and plants [15]. Arabidopsis mitochondrial basic amino acid carriers (AtmBAC1 and 2) transports Arg, Orn, lysine (Lys), and histidine (His) [15]. Consistently, their homologs, the Arg transporter *CsBAC2a* and Urea transporter *CsDUR3a* were also highly expressed in young leaves for mitochondria Arg-Orn degradation (Fig. 2C; Dataset S3, see online supplementary material).

Arg/Orn basic AA transporter UMAMIT genes were expressed in vascular tissues for xylem or phloem up- or down-loading of Arg and other AAs [51]. AtUMAMIT14 is an AA exporter involved in phloem unloading in Arabidopsis roots [52]. Their tea homolog genes *CsUmamiT11, 14, 18* were highly expressed in stems (Fig. 2C; Dataset S3, see online supplementary material). They also showed increasing transcript levels in young leaves in the top shoot of 8-year-old tea trees and *CsUMAMIT11a* and *14a* transcripts highly expressed in the stem internode S1 to S4 (Fig. 7E), where Arg was transported along xylem sap, and the transporters may facilitate Arg loading onto or downloading from xylem sap by AA transporters, such as CsLHT1 [52–55]. Functional characterization of tea plant CsCAT, CsBAT1, CsBAC1/2, CsUMAMIT14a, 11a, 18b, and CsAAP3 in future could provide more insights into AA transport mechanisms in tea plants.

### Integrating symplasmic and apoplastic paths for long-distance AA transport

During vegetative growth in perennial tea plants, developing leaves and young stems or roots are sinks for N; the root uptake and AA synthesis, translocation via xylem sap and unloading N from phloem, either in the form of NO_3_^−^, NH_4_^+^, or AAs, may follow the symplasmic and apoplastic path [56, 57]. N release from the phloem to the vegetative or reproductive sinks such as leaves, fruits, and seeds is generally via symplasmic movement through the plasmodesmata to the neighboring parenchyma cells [58]. AA and Urea transporters expressed in these tissues can function in re-cycling of organic N [3]. For instance, following symplasmic phloem unloading in ureides-transporting legumes, the major root-shoot N transport form ureides are degraded in the pod wall and seed coat cells and the released ammonia is re-assimilated into AAs [3, 59–63]. In soybean, ureides, mainly allantoin, and allantoic acid, are used for root-to-shoot transport of N in addition to AAs, because these ureide molecules have 4 N/4 C atom ratios as more efficient N transport form than Asn (2 N/4C) and Gln (2 N/5C) [3]. In tea plants, we demonstrated that Arg (4 N/6C) is the major root-shoot transport N form, and Arg is actively synthesized in roots, and transported to the phloem for uploading (Fig. 6). The long-distance transport of Arg, together with Glu, Gln and Thea, through xylem sap to the sink leaves and shoot tips, where Arg is degraded into Urea, Orn, and NH_3_ for re-assimilation and AA recycling (Fig. 9).

Dynamics of nitrogen uptake and assimilation and metabolism of major amino acids, such as Thea, Arg, Glu and Gln, and Urea in tea roots, long-distance transport of Arg, Glu, Gln, and Thea via xylem sap to the shoot tip, where young leaves have predominant Arg degradation and AA recycling into Gln and Thea, in which CsGS1a were critically involved. These metabolic pathways and enzyme activities in particular were enhanced in tea seedlings upon NH_4_^+^ supply.

Although Thea was mostly synthesized in roots by CsTSI and CsGSIa [26, 27], we observed that only a part of Thea was transported alongside with Arg, Glu, and Gln via root-shoot xylem sap to leaves. Despite consistent localization of CsGSIa and Thea in leaf veins with a recent report [37], our data suggest *de novo* Thea synthesis in local leaf mesophyll cells by CsGSIa, resembling Gln biosynthesis by GSs [11], which is consistent with the previous report [29, 60–63]. Furthermore, Thea could also be detected in phloem sap, indicating both up- and down-transport of Thea in tea stems [43]. Furthermore, whether or not Thea is also turned over during long-distance transport along xylem sap, like Arg and Gln, remains to be clarified.

Under N deficiency, tea roots showed a rapid decrease in Gln content, but slower decrease in Thea content [26], indicating the active Gln turnover for protein synthesis and AA metabolism, whereas much slower turnover for Thea as a specialized metabolite. So far, only a Thea hydrolase CsPDX2 was found for Thea degradation in EA and Glu [41], and data showed that *CsPDX2* was expressed higher in young leaves, basal stems, and roots, where Thea might be hydrolyzed (Fig. 1C and7E). Thus, Thea could act as a N reservoir in tea roots and shoot tips for use under special conditions. However, how Thea is loaded onto xylem sap in roots and downloaded from xylem sap to young leaf and shoot tips is also worthy of further investigation.

In summary, our study clarified the long-standing questions concerning root to shoot AA transport in tea plants. By dissecting AA metabolite and transcriptome variation across various tissues of tea plants, we demonstrated distinct features of AA metabolism in roots, stem, and leaves facilitating AA transport via xylem sap and NH_3_-re-assimilation and AA recycling in young leaves. It, furthermore, clarified that Arg, together with Glu, Gln, and Thea, are the major amino acids for long distance transport. These data enhance our understanding of the complexity of AA metabolism in tea plants. Furthermore, the study clarified the key roles of CsGSIa in N assimilation for Thea/Gln synthesis in roots, in long-distance AA transport in xylem sap, and NH_3_-re-assimilation in young leaves directly or indirectly. By doing so, the study provides comprehensive insights into AA metabolism and root to leaf transport in tea plants, potentially facilitating the alteration of AA production in tea leaves for improvement of tea flavor and health functions.

## Materials and methods

### Plant materials and growth conditions, treatment experiments

Tea plant (*C. sinensis* (L.) O. Kuntze ‘Longjing 43’) seedlings of 2 years old were grown hydroponically in Shigeki Konishi solution (SK medium). For N treatment experiments, two-year-old hydroponic tea seedlings were grown under long-day conditions (16 h/8 h of light/dark) at approximately 150 μmol photons m^−2^ s^−1^ during the light period at ~23°C until new tender roots emerged. The healthy tea seedlings were transferred into hydroponic SK medium supplemented with 5 mM ammonium N (final total N about 6.43 mM) or regular SK containing final total N as nearly 1.43 mM as a control (Table S1, see online supplementary material) [26].

The apical bud and first leaf (ST), first stem segment (SS1), second stem segment (SS2), third stem segment (SS3), fourth stem segment (SS4), and root samples of tea seedlings were collected and frozen in liquid nitrogen after treating for 10 days. For each treatment, we collected five different individual plants as biological duplication. The hydroponic solutions were replaced every five days. For dissection of tea roots, the lateral roots (LR), primary roots (PR), root-shoot junction (RSJ), lower stem segment 4 (LSS4), and higher stem segment 4 (HSS4) were sampled from 2-year-old hydroponically grown tea seedlings with newly developed tender roots treated or not treated with 5 mM NH_4_^+^ for 10 days and stored in liquid nitrogen rapidly. The top shoots of 8-year-old tea plants grown in tea garden were dissected into nine parts, from higher to lower positions, the first leaf, second leaf, third leaf, forth leaf, and fifth leaf, named as L1-L5, respectively, and the stem internodes between leaves from the top to lower position (removing any leaf and branch), named as S1 to S4, respectively.

### Extraction and quantification of free AAs

The free AAs in tea plant tissues were extracted from 120 mg tea tissues with 1 mL of 4% sulfosalicylic acid as described previously (Zhang *et al.*, 2021). The supernatants were filtered through a 0.22 μm Millipore filter before analysis. A mobile phase containing lithium citrate for AA derivatization and UV–Vis detection at 570 and 440 nm were used in the Hitachi High-Speed Amino Acid Analyzer system (L-8900, Hitachi) (Zhang *et al.*, 2021). The peak areas of AAs were quantified in comparison with the AA standards.

### Analysis of AAs in xylem sap, phloem, leaf petiole, vein, and leaf area tissues

The tea seedlings of Longjing 43 were hydroponically grown in SK media supplemented with or without 5 mM NH_4_^+^. After 3 days, seedlings were taken for excising various tissues, including roots, base xylem sap 3 (XS3) and phloem 3 (PL3), middle xylem sap 2 (XS2), and phloem 2 (PL2), old leaves, the top xylem sap 1 (XS1) and phloem 1 (PL1), and the young leaves for amino acid profiling. The tissues or xylem sap or phloem extrudes were extracted either by centrifugation of tissues or under vacuum suction according to the method described previously [64, 43]. Alternatively, absorbent cottons were used to cover top-pruned tea shoots and to absorb xylem saps under the natural root pressure for concentration and analysis of AAs [64]. The AAs in the extracts of the excised leaf petioles, veins, and leaf areas were analysed as mentioned above. The tea stem cross-sections were prepared from a Leica CM1900 vibrating blade microtome at −20°C. The 20 μm cross-sections in thickness were observed under a fluorescence microscope (Leica Microsystems DM3000) with 488 nm Ex for the auto-fluorescence of vascular tissues.

### RNA isolation, qRT-PCR analysis, and transcriptome sequencing

Total RNA was extracted by using an RNA extraction kit (Tiangen Biotech Co., Ltd., Beijing, China) and RNA quality was assessed by NanoDrop 2000 spectrophotometer (Thermo Scientific, Wilmington, DE, USA). The single-stranded cDNAs were synthesized by using SuperScript III reverse transcriptase for qRT-PCR analysis with the Prime Script RT Reagent Kit (TaKaRa, Dalian, China) on the Bio-Rad CFX96 fluorescence-based quantitative PCR platform, as previously described [65], with gene specific primers and reference genes *CsACTIN* listed in Table S2 (see online supplementary material). Transcriptome sequencing was performed by the Beijing Genome Institute (BGI, Shenzhen, China) as described previously [38, 37] (Li *et al.*, 2022a). The cDNA library was examined using an Agilent 2100 Bioanalyzer, then sequenced on an Illumina HiSeq 2500 sequencing platform. The raw data were analysed as previously described [65].

### *In situ* hybridization

*In situ* hybridization analysis of *CsGSIa* expression was performed as described by She *et al.* [26]. Gene-specific probes were synthesized and used for *in situ* hybridization as described in Table S2 (see online supplementary material).

### Knockdown and over-expression of *CsGSIa* in tea plants

Approximately 300 bp of the gene-specific fragments from *CsGSIa* were amplified and subcloned into the final RNA interference (RNAi) destination vector pB7GWIWG by BP and LR. The ORFs of *CsGSIa* was cloned into the entry vector pB2GW7 by BP and LR clonase-based recombination reactions (Invitrogen, USA) (Table S2, see online supplementary material). The selected positive *Agrobacterium rhizogenes* strain ATCC 15834 transformants harboring pB7GWIWG-*CsGSIa* and pB2GW7-*CsGSIa* were pretreated with acetosyringone and used to transform 3-month-old tea seedlings as described previously (Li *et al.*, 2022a; [26, 37]). For knockdown of *CsGSIa* genes in buds, an antisenseODN (*asODN*)-mediated suppression of *CsGSIa* in tea plant shoot tips was used, with sense ODN (*sODN*) as a control, according to the method described previously [38] (Table S2, see online supplementary material). Shoot tips were collected after treatment for 24, and 48 h for RNA analyses, Thea and Gln measurement, and enzyme activity assays, respectively [66].

### Statistical analysis

All experimental data were from at least three independent experiments. For transgenic hairy roots of *C. sinensis,* and antisense inhibition experiments, at least 10 independent transgenic lines were analysed. The differences in two-tailed tests of data group comparisons with the error bars marked represent 95% confidence intervals.

## Supplementary Material

Web_Material_uhae060

## Data Availability

The data of this article that support the findings of this study are available in the supplementary materials, including Tables S1–S2, Datasets S1–S9, and Tea Plant Information Archive (TPIA; http://tpia.teaplant.org), and are publicly accessible. Other materials and information will be available upon request from the corresponding author, who will be also responsible for distribution of materials integral to the findings presented in this article in accordance with the policy described in the Instructions for Authors: Jian Zhao (jzhao2@qq.com; zhaojian@hunau.edu.cn).
