# Supplementary material for: Dissection of the spatial dynamics of biosynthesis, transport, and turnover of major amino acids in tea plants (Camellia sinensis)
Source: Hortic Res. 2024 Feb 19;11(5):uhae060. doi: 10.1093/hr/uhae060 (PMC11070726; doi:10.1093/hr/uhae060)
Supplement: Web_Material_uhae060 [file web_material_uhae060.zip › Supplemental Figures S1-S5.pdf]

Supplemental Figure S1 :

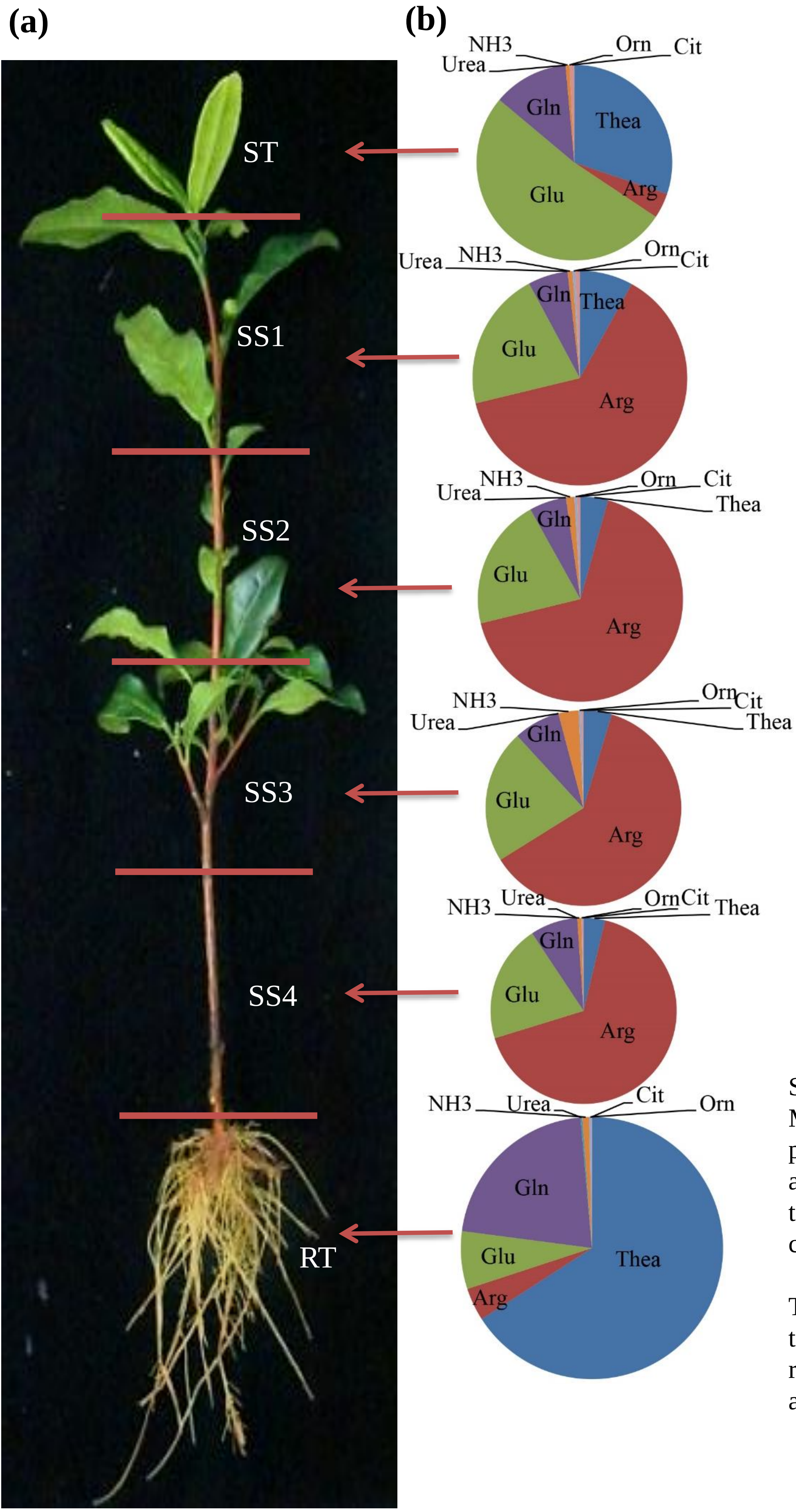

Supplemental Figure S1 :
Major amino acids for their proportions in whole free amino acids and N-metabolites of six tissues under normal nitrogen condition.

The sizes of these pies represented total amino acid contents, slices represented different major amino acids.

Supplemental Figure S2 :

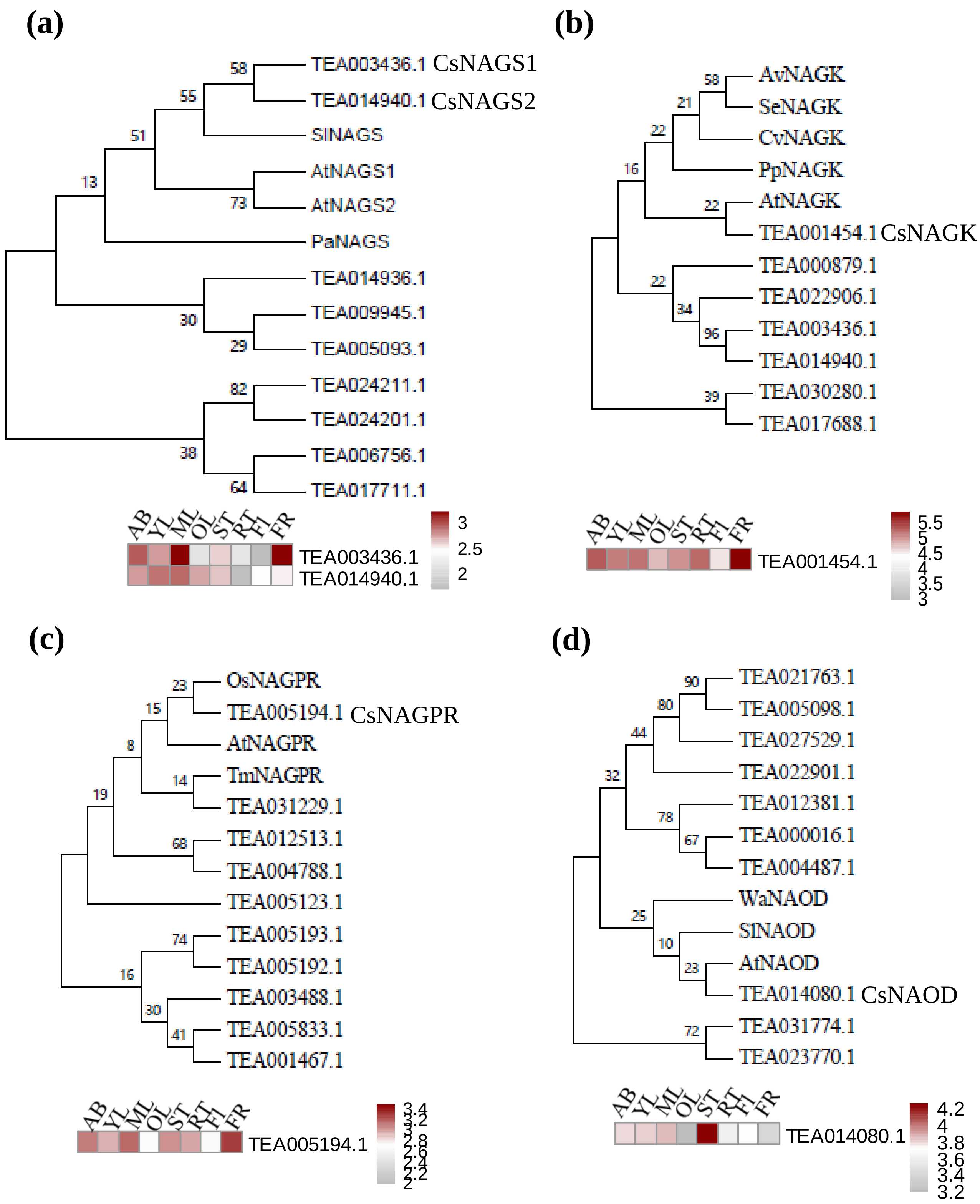

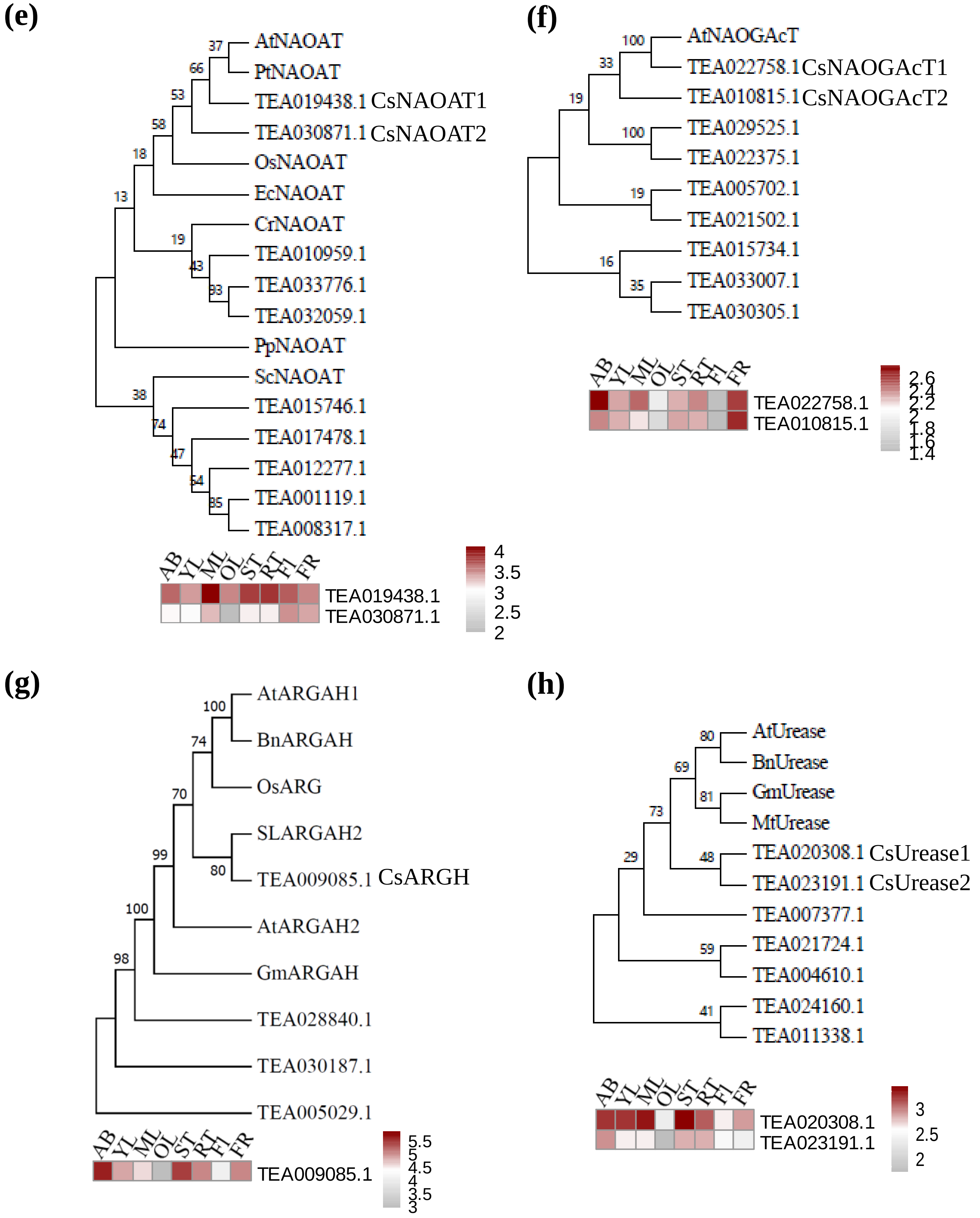
(e)
AtNAOAT
PtNAOAT
TEA019438.1 CsNAOAT1
TEA030871.1 CsNAOAT2
OsNAOAT
EcNAOAT
CrNAOAT
TEA010959.1
TEA033776.1
TEA032059.1
PpNAOAT
ScNAOAT
TEA015746.1
TEA017478.1
TEA012277.1
TEA001119.1
TEA008317.1
AB YL ML OL ST RT FI FR
TEA019438.1
TEA030871.1
4
3.5
3
2.5
2
(f)
AtNAOGAcT
TEA022758.1 CsNAOGAcT1
TEA010815.1 CsNAOGAcT2
TEA029525.1
TEA022375.1
TEA005702.1
TEA021502.1
TEA015734.1
TEA033007.1
TEA030305.1
AB YL ML OL ST RT FI FR
TEA022758.1
TEA010815.1
2.6
2.4
2.2
2
1.8
1.6
1.4
(g)
AtARGAH1
BnARGAH
OsARG
SLARGAH2
TEA009085.1 CsARGH
AtARGAH2
GmARGAH
TEA028840.1
TEA030187.1
TEA005029.1
AB YL ML OL ST RT FI FR
TEA009085.1
5.5
5
4.5
4
3.5
3
(h)
AtUrease
BnUrease
GmUrease
MtUrease
TEA020308.1 CsUrease1
TEA023191.1 CsUrease2
TEA007377.1
TEA021724.1
TEA004610.1
TEA024160.1
TEA011338.1
AB YL ML OL ST RT FI FR
TEA020308.1
TEA023191.1
3
2.5
2

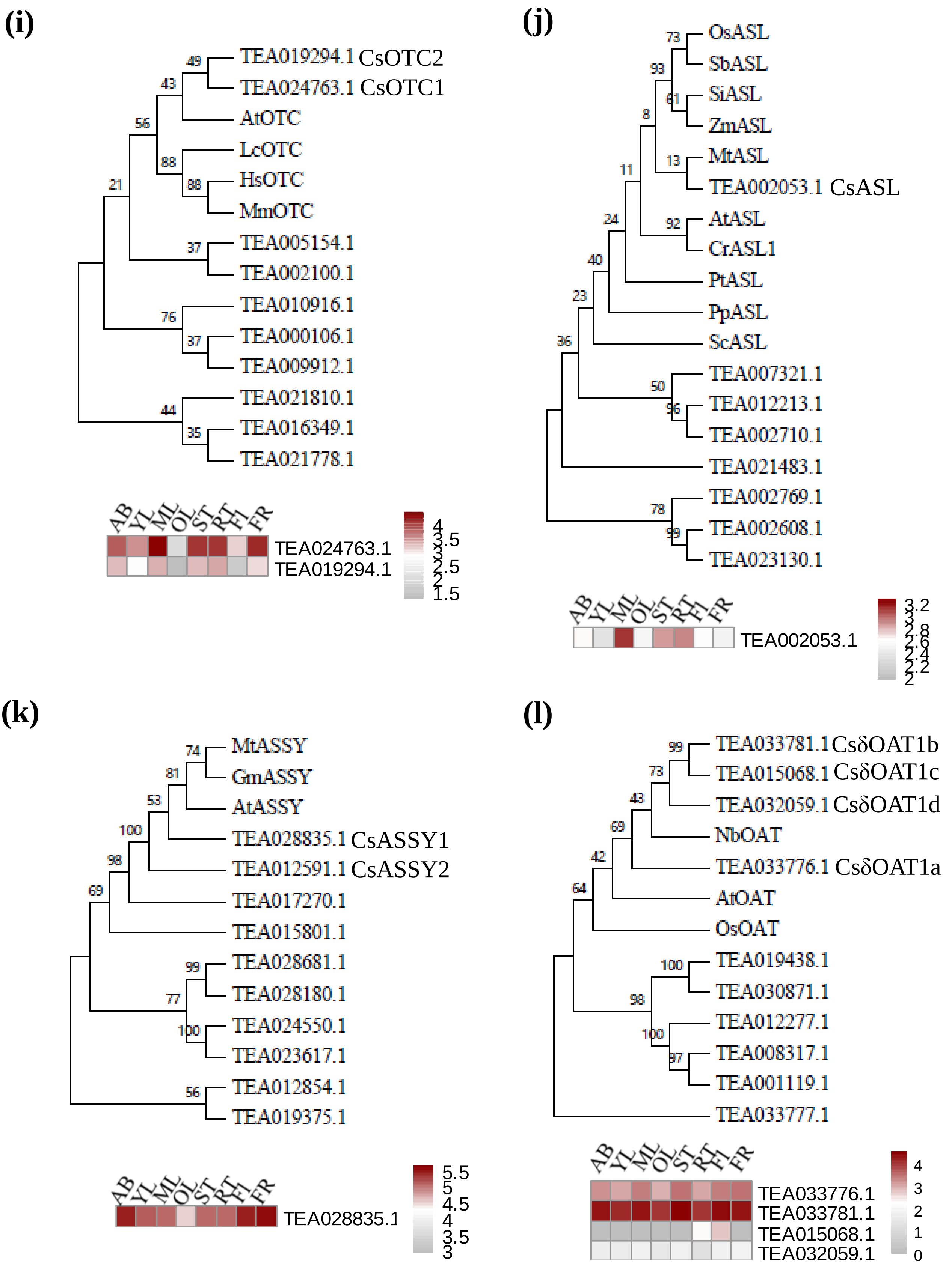
(i)
TEA019294.1 CsOTC2
TEA024763.1 CsOTC1
AtOTC
LcOTC
HsOTC
MmOTC
TEA005154.1
TEA002100.1
TEA010916.1
TEA000106.1
TEA009912.1
TEA021810.1
TEA016349.1
TEA021778.1
AB YL ML OL ST RT FL FR
TEA024763.1
TEA019294.1
4 3.5 3 2.5 2 1.5
(j)
OsASL
SbASL
SiASL
ZmASL
MtASL
TEA002053.1 CsASL
AtASL
CrASL1
PtASL
PpASL
ScASL
TEA007321.1
TEA012213.1
TEA002710.1
TEA021483.1
TEA002769.1
TEA002608.1
TEA023130.1
AB YL ML OL ST RT FL FR
TEA002053.1
3.2 3 2.8 2.6 2.4 2.2 2
(k)
MtASSY
GmASSY
AtASSY
TEA028835.1 CsASSY1
TEA012591.1 CsASSY2
TEA017270.1
TEA015801.1
TEA028681.1
TEA028180.1
TEA024550.1
TEA023617.1
TEA012854.1
TEA019375.1
AB YL ML OL ST RT FL FR
TEA028835.1
5.5 5 4.5 4 3.5 3
(l)
TEA033781.1 CsδOAT1b
TEA015068.1 CsδOAT1c
TEA032059.1 CsδOAT1d
NbOAT
TEA033776.1 CsδOAT1a
AtOAT
OsOAT
TEA019438.1
TEA030871.1
TEA012277.1
TEA008317.1
TEA001119.1
TEA033777.1
AB YL ML OL ST RT FL FR
TEA033776.1
TEA033781.1
TEA015068.1
TEA032059.1
4 3 2 1 0

(m)

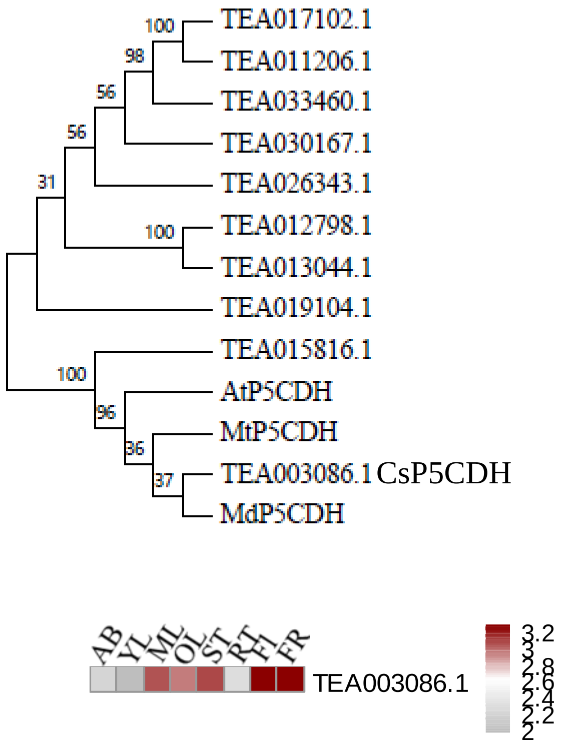
TEA017102.1
TEA011206.1
TEA033460.1
TEA030167.1
TEA026343.1
TEA012798.1
TEA013044.1
TEA019104.1
TEA015816.1
AtP5CDH
MtP5CDH
TEA003086.1 CsP5CDH
MdP5CDH
AB YL ML OL ST RT FL FR
TEA003086.1
3.2
3
2.8
2.6
2.4
2.2
2

(n)

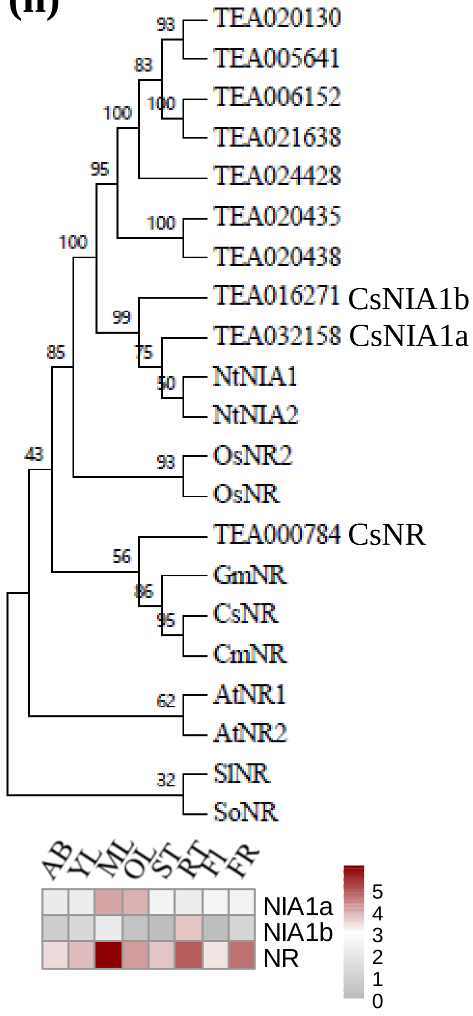
TEA020130
TEA005641
TEA006152
TEA021638
TEA024428
TEA020435
TEA020438
TEA016271 CsNIA1b
TEA032158 CsNIA1a
NtNIA1
NtNIA2
OsNR2
OsNR
TEA000784 CsNR
GmNR
CsNR
CmNR
AtNR1
AtNR2
SlNR
SoNR
AB YL ML OL ST RT FL FR
NIA1a
NIA1b
NR
5
4
3
2
1
0

(o)

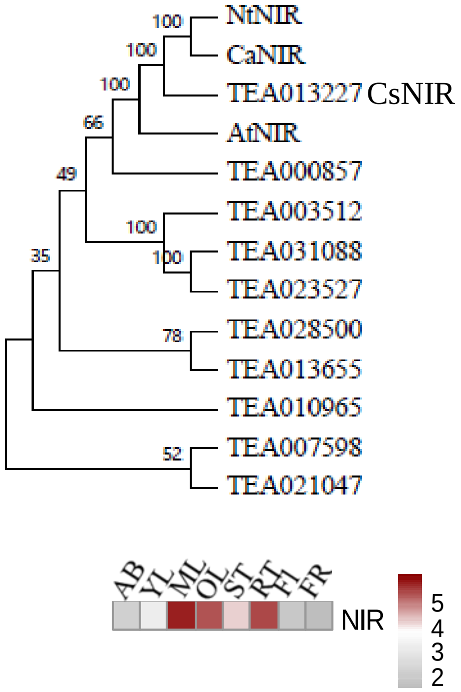
NtNIR
CaNIR
TEA013227 CsNIR
AtNIR
TEA000857
TEA003512
TEA031088
TEA023527
TEA028500
TEA013655
TEA010965
TEA007598
TEA021047
AB YL ML OL ST RT FL FR
NIR
5
4
3
2

(p)

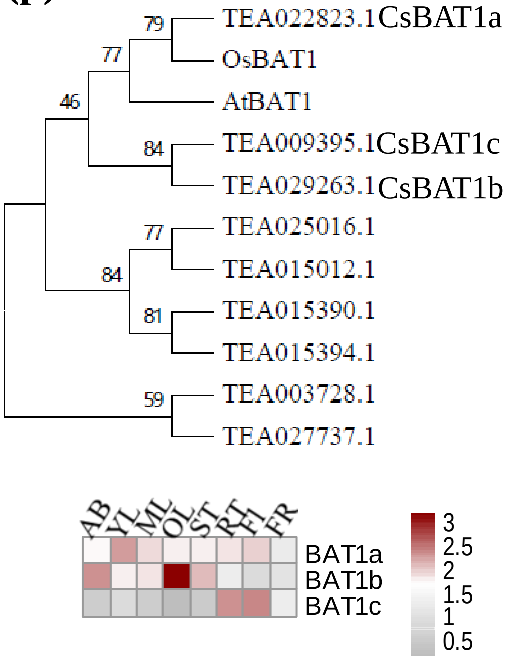
TEA022823.1 CsBAT1a
OsBAT1
AtBAT1
TEA009395.1 CsBAT1c
TEA029263.1 CsBAT1b
TEA025016.1
TEA015012.1
TEA015390.1
TEA015394.1
TEA003728.1
TEA027737.1
AB YL ML OL ST RT FL FR
BAT1a
BAT1b
BAT1c
3
2.5
2
1.5
1
0.5

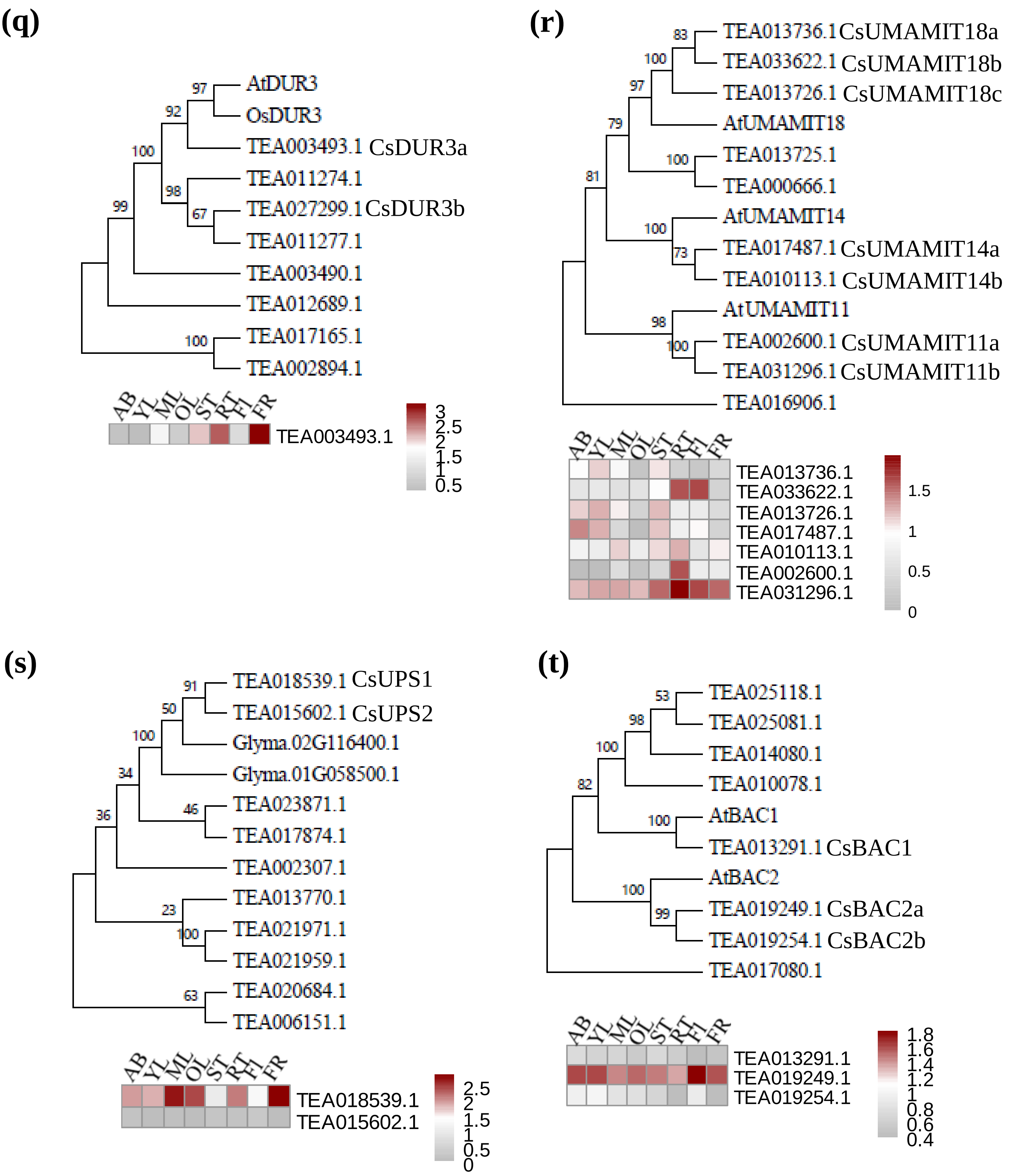

(q)
AtDUR3
OsDUR3
TEA003493.1 CsDUR3a
TEA011274.1
TEA027299.1 CsDUR3b
TEA011277.1
TEA003490.1
TEA012689.1
TEA017165.1
TEA002894.1
97
92
100
98
67
99
100
AB YL ML OL ST RT FI FR
TEA003493.1
3
2.5
2
1.5
1
0.5
(r)
TEA013736.1 CsUMAMIT18a
TEA033622.1 CsUMAMIT18b
TEA013726.1 CsUMAMIT18c
AtUMAMIT18
TEA013725.1
TEA000666.1
AtUMAMIT14
TEA017487.1 CsUMAMIT14a
TEA010113.1 CsUMAMIT14b
AtUMAMIT11
TEA002600.1 CsUMAMIT11a
TEA031296.1 CsUMAMIT11b
TEA016906.1
83
100
97
79
100
81
100
73
98
100
AB YL ML OL ST RT FI FR
TEA013736.1
TEA033622.1
TEA013726.1
TEA017487.1
TEA010113.1
TEA002600.1
TEA031296.1
1.5
1
0.5
0
(s)
TEA018539.1 CsUPS1
TEA015602.1 CsUPS2
Glyma.02G116400.1
Glyma.01G058500.1
TEA023871.1
TEA017874.1
TEA002307.1
TEA013770.1
TEA021971.1
TEA021959.1
TEA020684.1
TEA006151.1
91
50
100
34
46
36
23
100
63
AB YL ML OL ST RT FI FR
TEA018539.1
TEA015602.1
2.5
2
1.5
1
0.5
0
(t)
TEA025118.1
TEA025081.1
TEA014080.1
TEA010078.1
AtBAC1
TEA013291.1 CsBAC1
AtBAC2
TEA019249.1 CsBAC2a
TEA019254.1 CsBAC2b
TEA017080.1
53
98
100
82
100
100
99
AB YL ML OL ST RT FI FR
TEA013291.1
TEA019249.1
TEA019254.1
1.8
1.6
1.4
1.2
1
0.8
0.6
0.4

(u)

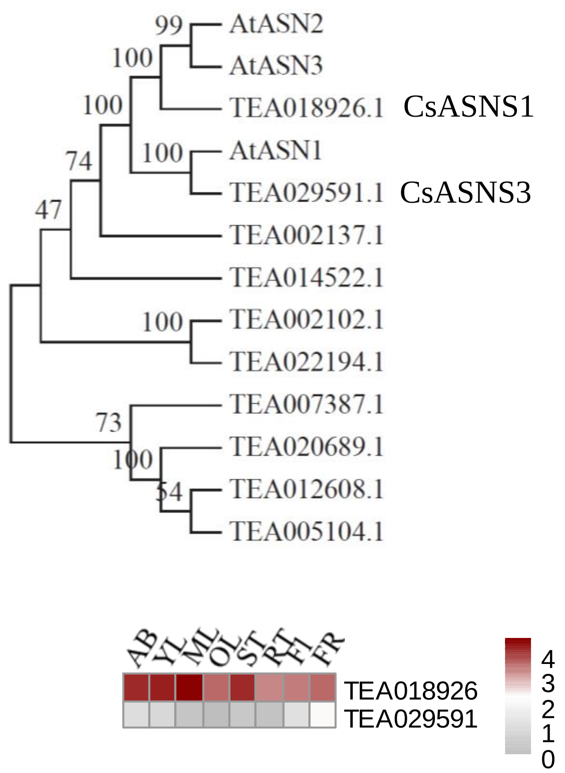

(v)

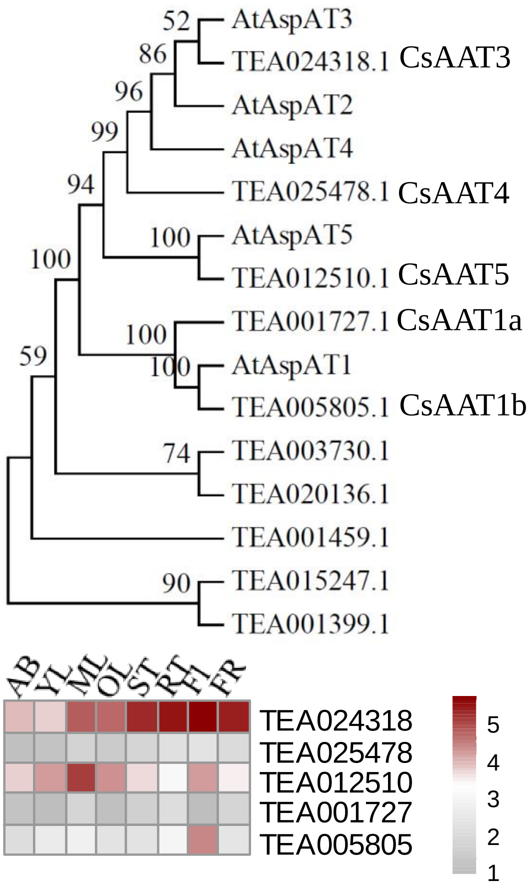

Supplemental Figure S2: The phylogenetic analyses and expression patterns of genes involved N uptake and assimilation, AA metabolism and transport in tea plants.

The functionally characterized *Arabidopsis*, *Oryza sativa*, and *soybean* homologue proteins were used as references.
Amino acid sequences were aligned by using Clustal W, and MEGA 6.0 software was used to construct the phylogenetic tree by the NJ method with 1000 bootstrap replicates. Expression of genes in different tissues of Camellia sinensis plants were retrieved from.

Supplemental Figure S3 :

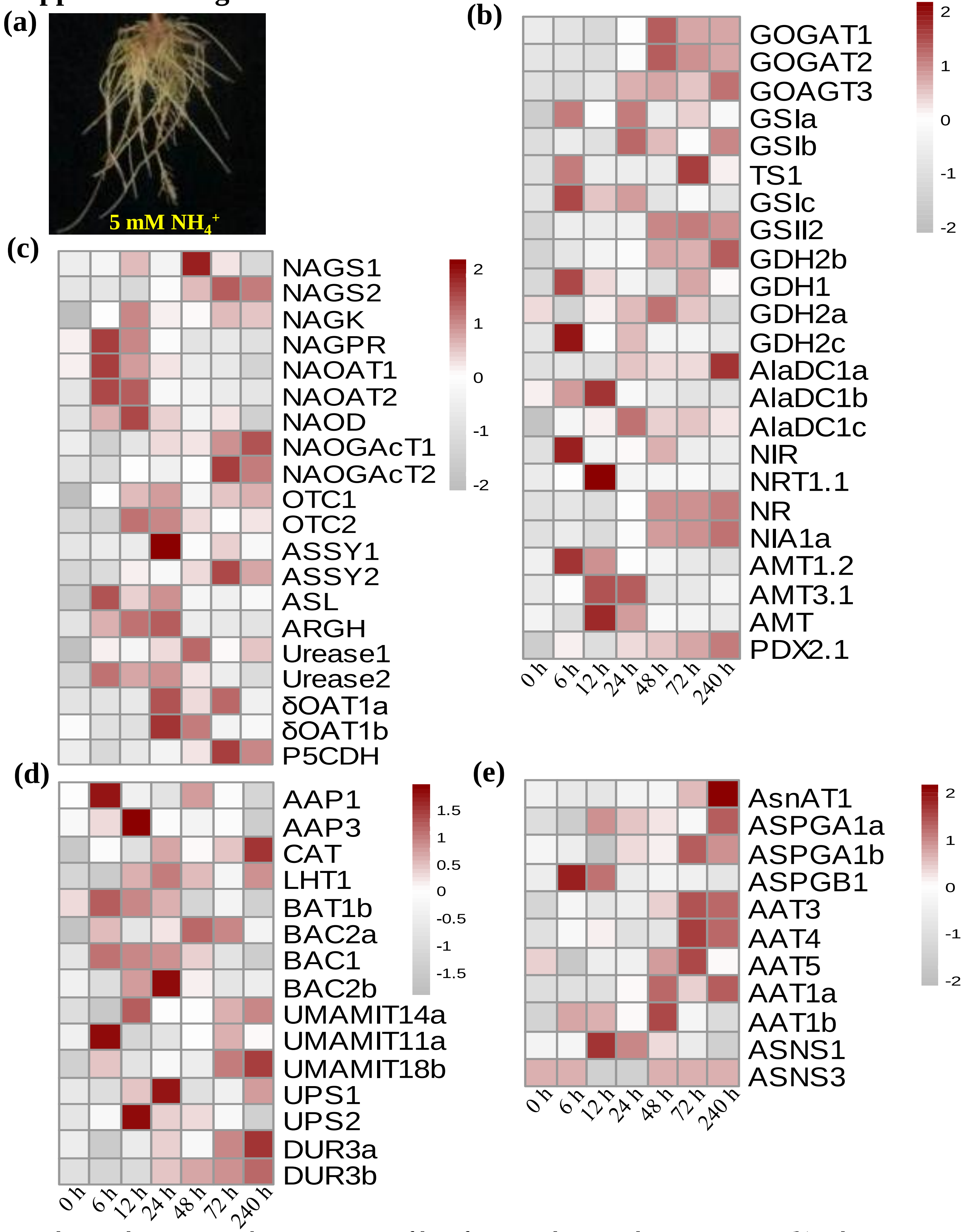

Supplemental Figure S3: The expression profiles of N assimilation and transport genes (b), Glu-Arg-Urea synthesis and degradation pathways genes (c), and amino acid transport genes (d), Asp-Glu synthesis and degradation pathways genes (e) in the root of tea plant seedlings fed with 5 mM NH_4+.

Supplemental Figure S4 :

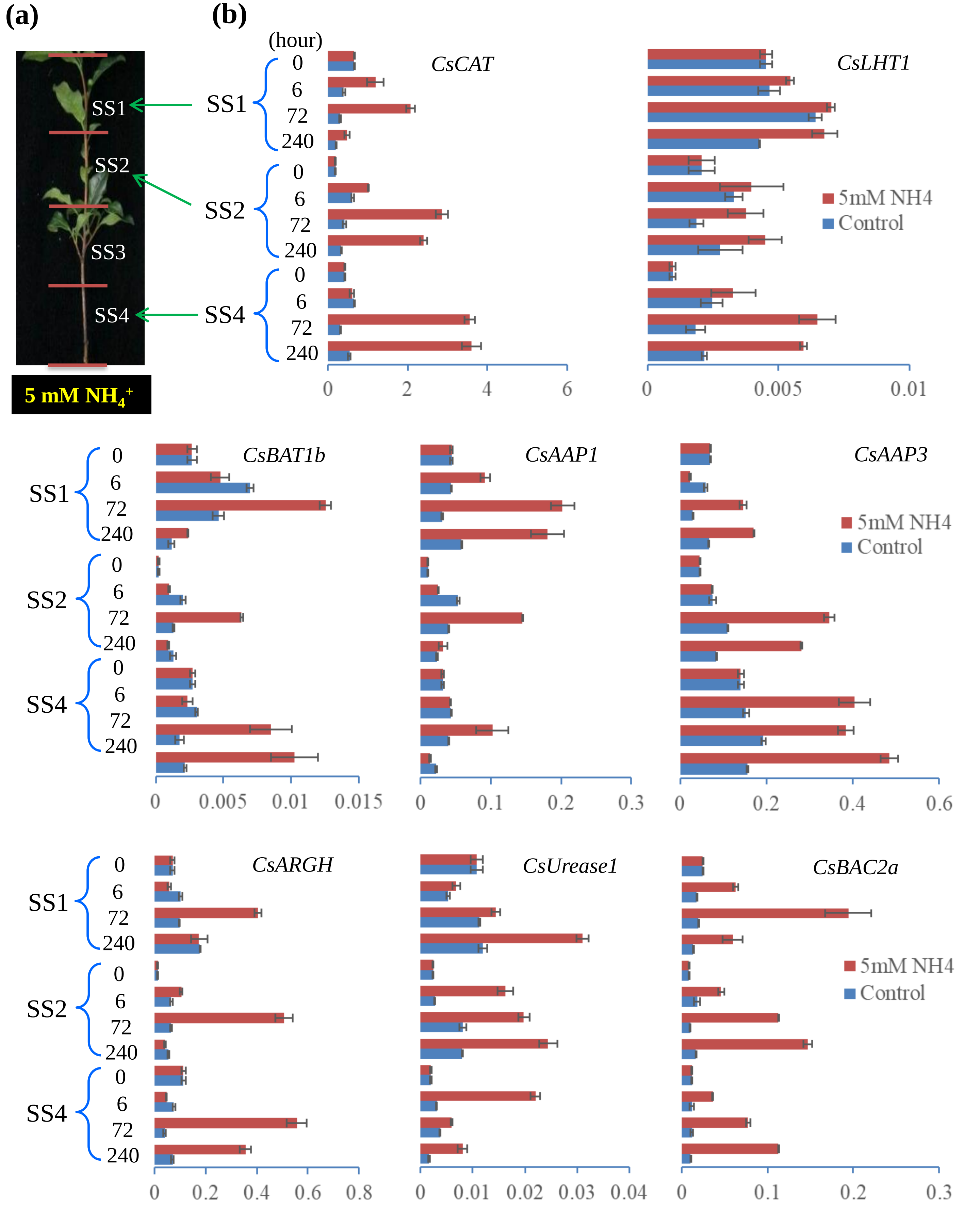

Supplemental Figure S4: Q-pcr analysis expression patterns of several amino acid transport and metabolic genes in stems of various fragments of tea plant seedlings fed with 5 mM NH4+.

Supplemental Figure S5 :

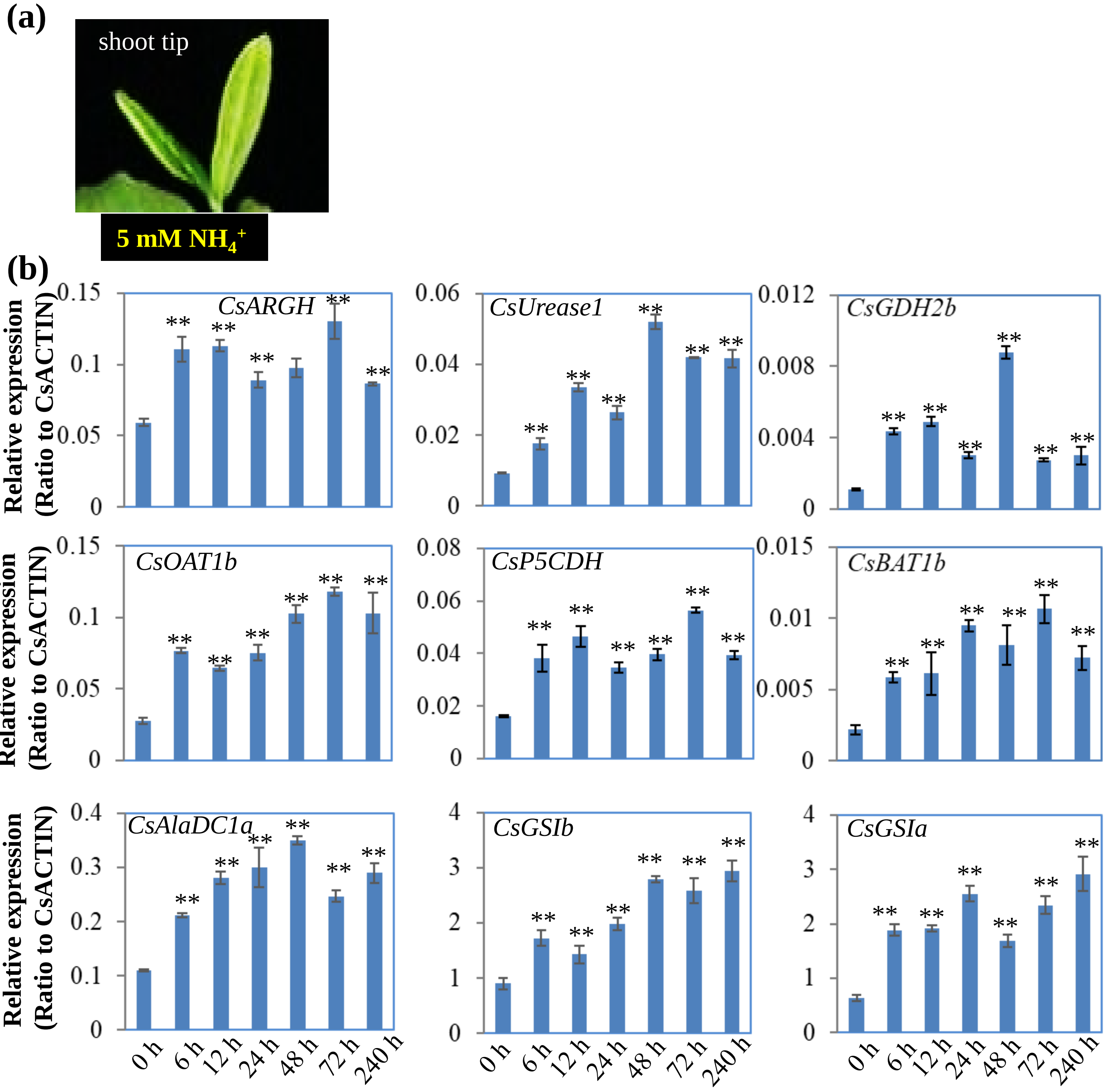

Supplemental Figure S5: Expression profiles of genes involved in Arg-Urea synthesis, Arg-Orn-Glu degradation, Thea/Gln synthesis, and AA transport processes in the apical bud and the first young leaf of tea plant seedlings fed with 5 mM NH_4+.
